# Tackling visual impairment: emerging avenues in ophthalmology

**DOI:** 10.3389/fmed.2025.1567159

**Published:** 2025-04-28

**Authors:** Fang Lin, Yuxing Su, Chenxi Zhao, Farhana Akter, Shun Yao, Sheng Huang, Xiaodong Shao, Yizheng Yao

**Affiliations:** ^1^Department of Ophthalmology, Xinjiang 474 Hospital, China RongTong Medical Healthcare Group CO. LTD, Urumqi, Xinjiang Uygur Autonomous Region, China; ^2^Faculty of Arts and Sciences, Harvard University, Cambridge, MA, United States; ^3^Department of Neurosurgery, The First Affiliated Hospital, Sun Yat-sen University, Guangzhou, Guangdong, China; ^4^Department of Ophthalmology, TongRen Municipal People’s Hospital, Tongren, Guizhou, China; ^5^Department of Neurology and Clinical Research Center of Neurological Disease, The Second Affiliated Hospital of Soochow University, Soochow University, Suzhou, Jiangsu, China

**Keywords:** gene therapy, stem cell therapy, nanotechnology, artificial intelligence, teleophthalmology, optogenetics, bionics, neuro-ophthalmology

## Abstract

Visual impairment, stemming from genetic, degenerative, and traumatic causes, affects millions globally. Recent advancements in ophthalmology present novel strategies for managing and potentially reversing these conditions. Here, we explore 10 emerging avenues—including gene therapy, stem cell therapy, advanced imaging, novel therapeutics, nanotechnology, artificial intelligence (AI) and machine learning, teleophthalmology, optogenetics, bionics, and neuro-ophthalmology—all making strides to improve diagnosis, treatment, and vision restoration. Among these, gene therapy and stem cell therapy are revolutionizing the treatment of retinal degenerative diseases, while advanced imaging technologies enable early detection and personalized care. Therapeutic advancements like anti-vascular endothelial growth factor therapies and neuroprotective agents, along with nanotechnology, have improved clinical outcomes for multiple ocular conditions. AI, especially machine learning, is enhancing diagnostic accuracy, facilitating early detection, and personalized treatment strategies, particularly when integrated with advanced imaging technologies. Teleophthalmology, further strengthened by AI, is expanding access to care, particularly in underserved regions, whereas emerging technologies like optogenetics, bionics, and neuro-ophthalmology offer new hope for patients with severe vision impairment. In light of ongoing research, we summarize the current clinical landscape and the potential advantages of these innovations to revolutionize the management of visual impairments. Additionally, we address the challenges and limitations associated with these emerging avenues in ophthalmology, providing insights into their future trajectories in clinical practice. Continued advancements in these fields promise to reshape the landscape of ophthalmic care, ultimately improving the quality of life for individuals with visual impairments.

## 1 Introduction

**Figure 1:**
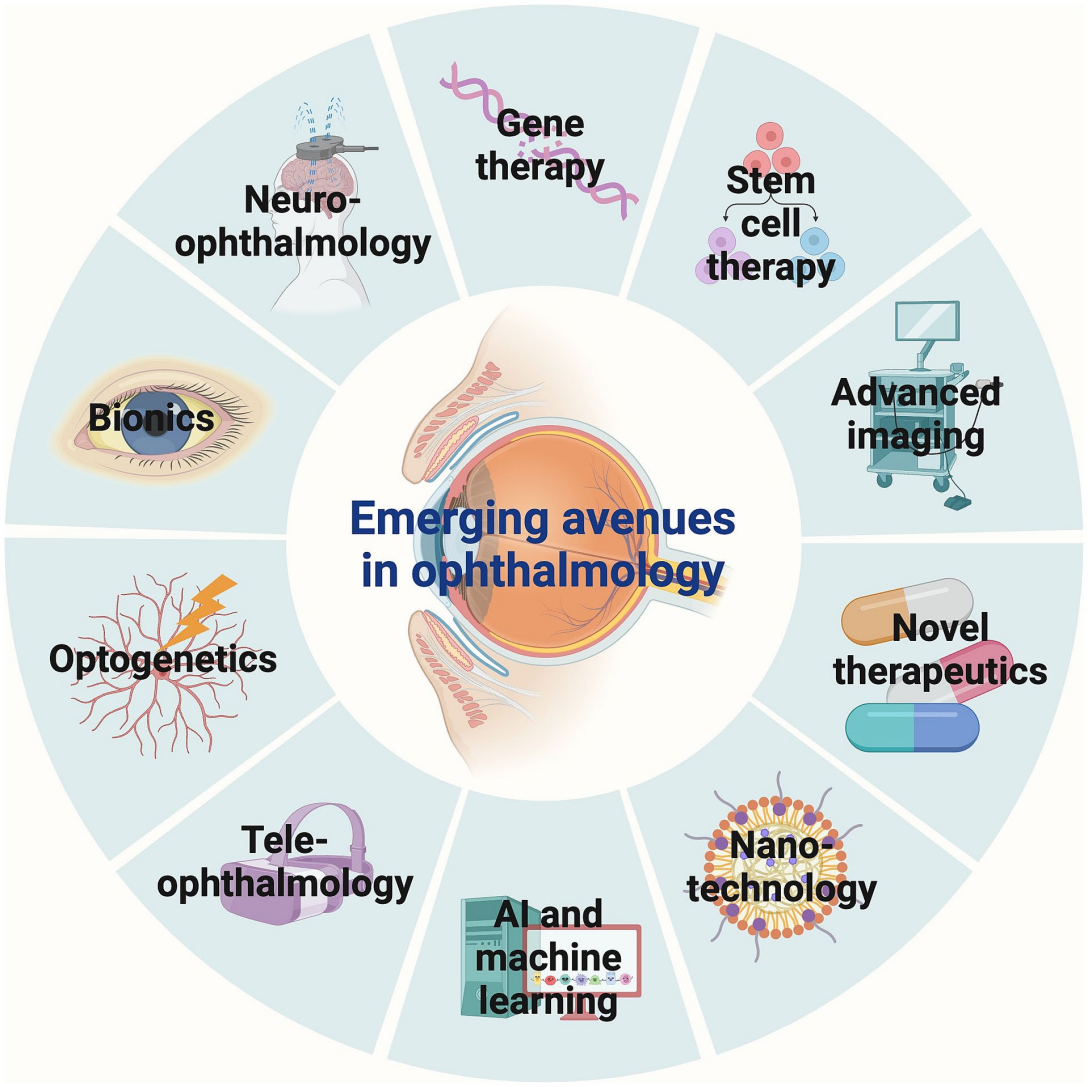
Emerging avenues in ophthalmology. Gene therapy, stem cell therapy, advanced imaging, novel therapeutics, nanotechnology, AI and machine learning, teleophthalmology, optogenetics, bionics, and neuro-ophthalmology represent 10 key areas of advancements in ophthalmology. AI, Artificial intelligence.

Visual impairment remains a significant global health challenge, affecting over 1 billion people worldwide. It encompasses a wide range of etiologies, from congenital genetic disorders to acquired conditions such as age-related macular degeneration (AMD) and diabetic retinopathy, resulting in partial vision loss to total blindness (1, 2). Traditional treatments have predominantly focused on symptom management, often leaving the underlying causes of vision loss unaddressed (3). However, the field of ophthalmology is undergoing a profound transformation, marked by groundbreaking therapeutic innovations, advanced diagnostic technologies, and the integration of cutting-edge fields such as gene therapy, stem cell therapy, nanotechnology, and artificial intelligence (AI), as well as emerging interdisciplinary approaches that promise to redefine visual restoration and care (4–7). These innovations have the potential not only to halt the progression of visual impairment but also to restore vision in conditions previously considered untreatable (3, 4). This review provides a comprehensive overview of 10 key areas of advancement in ophthalmology: gene therapy, stem cell therapy, advanced imaging, novel therapeutics, nanotechnology, AI and machine learning, teleophthalmology, optogenetics, bionics, and neuro-ophthalmology (Figure 1). Each of these areas represents a significant leap forward in our ability to diagnose, treat, and potentially cure various forms of visual impairment and loss.

## 2 Gene therapy

**Figure 2:**
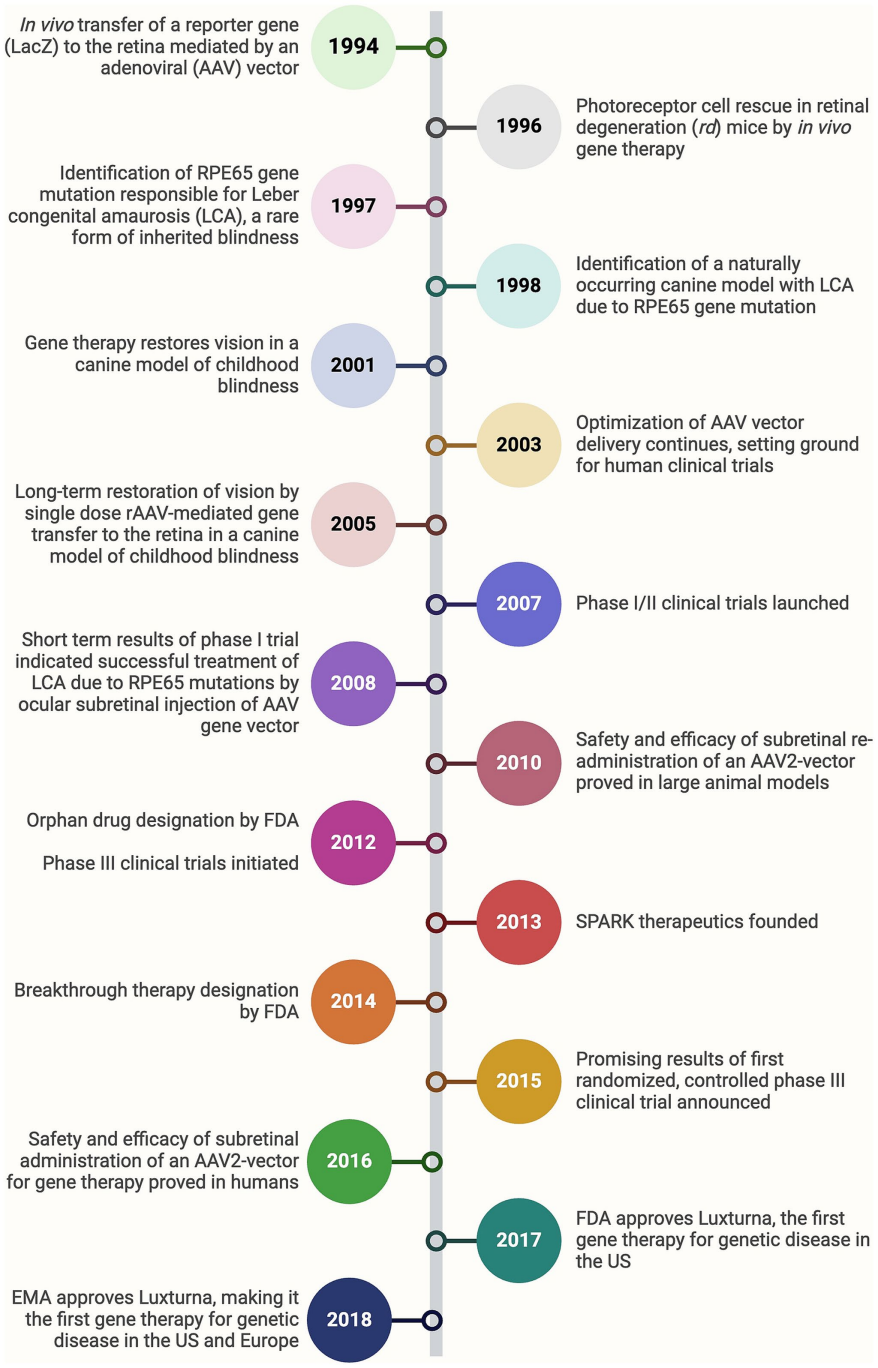
Bench-to-bedside timeline of the development of Luxturna, the first-ever gene therapy approved for a genetic disease. FDA, Food and drug administration; EMA, European medicine agency.

Gene therapy has emerged as a groundbreaking approach for treating inherited retinal diseases caused by mutations in specific genes. These disorders, including retinitis pigmentosa, Leber congenital amaurosis (LCA), and achromatopsia, often lead to progressive vision loss and, in many cases, blindness (8). The underlying principle of gene therapy is to introduce functional copies of defective genes into affected cells, thereby correcting the genetic defect and restoring normal cellular function (9). One of the most significant developments in gene therapy for ophthalmology is the use of adeno-associated viruses (AAVs) as vectors to deliver therapeutic genes into retinal cells. AAVs are preferred due to their ability to effectively transduce retinal cells and their relatively low immunogenicity (10). The success of gene therapy in ophthalmology was exemplified by the Food and Drug Administration (FDA) approval of Luxturna® (voretigene neparvovec-rzyl) in 2017, the first gene therapy approved for an inherited disease (Figure 2) (11, 12). Luxturna® delivers a functional copy of the retinal pigment epithelium-specific 65 (RPE65) gene to the retinal pigment epithelium (RPE) cells of patients with LCA, leading to significant improvements in vision (13). Beyond RPE65, gene therapy is being explored for various other genetic eye conditions. For example, ongoing clinical trials are targeting the cyclic nucleotide-gated cation channel alpha-3 (CNGA3) and beta-3 (CNGB3) genes responsible for achromatopsia, with early results indicating safety and potential efficacy (14). Similarly, therapies targeting the vascular endothelial growth factor (VEGF) gene for wet AMD aim to reduce the frequency of anti-VEGF injections, which are currently the standard treatment. These trials utilize AAV vectors to deliver genes that produce anti-VEGF proteins within the eye, potentially providing a long-lasting therapeutic effect (15). For instance, Phase I/IIa of RGX-314 has been completed, with ongoing long-term follow-up. The study has shown that subretinal delivery of RGX-314 via a transvitreal approach is generally well-tolerated, without unusual immune responses or ocular inflammation (15, 16). Another area of active research is gene therapy for X-linked retinitis pigmentosa, a condition caused by mutations in the retinitis pigmentosa GTPase regulator (RPGR) gene. Initial trials using AAV vectors to deliver a functional RPGR gene have shown promising results, with some patients experiencing stabilization of their vision (17). A comprehensive list of clinical studies assessing different gene therapies in ophthalmology is provided in Table 1.

**Table 1:** Clinical studies assessing different gene therapies in ophthalmology.

NCT ID	Condition	Product	Gene target	Vector	Clinical phase	Outcome
NCT02935517	Achromatopsia	AGTC-402	CNGA3	AAV2	Phase I/II	Ongoing, safety established, assessing visual acuity
NCT03758404	Achromatopsia	AAV-CNGA3	CNGA3	AAV8	Phase I/II	Ongoing, early safety data suggests tolerability, visual function assessments ongoing
NCT02610582	Achromatopsia	rAAV2-hCNGA3	CNGA3	AAV2	Phase I/II	Ongoing, preliminary results show safety and potential for restoring some visual function
NCT03278873	Achromatopsia	MeiraGTx-A002	CNGB3	AAV8	Phase I/II	Ongoing, evaluating safety and efficacy, preliminary data indicate potential visual improvements
NCT03001310	Achromatopsia	AAV-CNGB3	CNGB3	AAV8	Phase I/II	Ongoing, safety profile being monitored, with assessments on light perception and visual acuity
NCT02599922	Achromatopsia	AGTC-401	CNGB3	AAV2	Phase I/II	Ongoing, similar to AGTC-402
NCT03920007	Autosomal recessive LCA	SAR439483	GUCY2D	AAV5	Phase I/II	Ongoing, initial results promising in safety profile
NCT04483440	Choroideremia	4D-110	REP1	Capsid Variant 4D-R100	Phase I	Ongoing, initial safety profile established
NCT03584165	Choroideremia	BIIB111	REP1	AAV2	Phase III	Positive results in slowing disease progression, some vision improvement
NCT04418427	DME	ADVM-022	VEGF	AAV.7m8	Phase II	Ongoing, assessing safety and efficacy
NCT04567550 NCT05296447	Diabetic retinopathy	RGX-314	Anti-VEGF Fab	AAV8	Phase II	Ongoing, early results show potential efficacy
NCT03846193	Dry AMD	GT005	Complement Factor H (CFH)	rAAV	Phase I/II	Ongoing, preliminary data suggest reduced drusen area
NCT04435366 NCT05536297	Geographic atrophy	Zimura (avacincaptad pegol)	C5	NA (aptamer)	Phase III	Phase II showed reduced progression of geographic atrophy
NCT02161380	Leber hereditary optic neuropathy	scAAV2-P1ND4v2	ND4	AAV2	Phase I	Ongoing, assessing safety and potential vision improvement
NCT03293524	Leber hereditary optic neuropathy	GS010	ND4	rAAV2/2	Phase III	Phase III results pending, initial trials showed some efficacy
NCT03872479	LCA	EDIT-101	CEP290	Gene editing via CRISPR/Cas9	Phase I/II	Ongoing, initial safety and feasibility established with improved vision in some patients
NCT01208389 NCT03597399 NCT00999609 NCT03602820	LCA	AAV2-hRPE65v2	RPE65	AAV2	Phase I/II; III; follow-up	Significant vision improvement in some patients, ongoing follow-up
NCT02946879	LCA	AAV2/5-OPTIRPE65	RPE65	AAV2/5	Follow-up	Follow-up, monitoring long-term safety and efficacy
NCT03326336	Non-syndromic retinitis pigmentosa	GS030-DP with medical device GS030-MD	Channelrhodopsin-2 (ChR2)	AAV2	Phase I/II	Early-phase, assessing safety and efficacy
NCT03328130	Retinitis pigmentosa	AAV2/5-hPDE6B	PDE6B	AAV2/5	Phase I/II	Ongoing, initial safety and efficacy data positive
NCT04278131	Retinitis pigmentosa	BS01	Channelrhodopsin-2 (ChR2)	rAAV	Phase I/II	Ongoing, safety profile being established
NCT04611503	Retinitis pigmentosa	rAAV.hPDE6A	PDE6A	rAAV	Phase I/II	Ongoing, assessing safety and initial efficacy
NCT05203939	Retinitis pigmentosa	OCU400	NR2E3, RHO	AAV5	Phase I/II	Ongoing, assessing safety and initial efficacy
NCT04945772	Retinitis pigmentosa	vMCO-010	MCO1	AAV2/5	Phase II	Early-phase, assessing safety and efficacy
NCT05085964	Retinitis pigmentosa	QR 421a	Exon 13 of USH2A	RNA antisense oligonucleotide	Phase II	Phase II ongoing, promising early results
NCT03364153	Stargardt disease	Zimura (avacincaptad pegol)	C5	NA (aptamer)	Phase II	Ongoing, phase II showed reduced progression of macular atrophy
NCT05417126	Stargardt disease	vMCO-010	MCO1	AAV2	Phase II	Early-phase, assessing safety and efficacy
NCT05197270	Wet AMD	4D-150	VEGF	AAV	Phase I/II	Early-phase, assessing safety and initial efficacy
NCT04704921	Wet AMD	ABBV-RGX-314	Anti-VEGF Fab	AAV8	Phase II/III	Ongoing, evaluating safety, tolerability, and dose response; early data suggests reduced need for anti-VEGF injections
NCT05407636	Wet AMD	ABBV-RGX-314	Anti-VEGF Fab	AAV8	Phase III	Ongoing, assessing efficacy in reducing the frequency of anti-VEGF injections and improving visual acuity
NCT04514653	Wet AMD	ABBV-RGX-314	Anti-VEGF Fab	AAV8	Phase II	Ongoing, initial results indicate a positive safety profile and potential reduction in treatment burden
NCT04832724	Wet AMD	RGX-314	Anti-VEGF Fab	AAV8	Phase II/III	Positive interim results, reduction in anti-VEGF injections
NCT05536973	Wet AMD	ADVM-022	VEGF	AAV.7m8	Phase II	Ongoing, shows promise in reducing VEGF levels
NCT03316560	X-linked retinitis pigmentosa	AGTC-501	RPGR	rAAV2	Phase I/II	Ongoing, initial results positive
NCT04517149	X-linked retinitis pigmentosa	4D-125	RPGR	Capsid Variant 4D-R100	Phase I/II	Early-phase, assessing safety and initial efficacy
NCT03584165	X-linked retinitis pigmentosa	BIIB112	RPGR	AAV8	Phase III	Positive results in slowing disease progression, some vision improvement
NCT04671433 NCT04794101	X-linked retinitis pigmentosa	AAV5-RPGR	RPGR	AAV5	Phase III	Ongoing, promising initial results in some patients
NCT02317887	X-linked retinoschisis	AAV-RS1	RS1	AAV8	Phase I/II	Early-phase, assessing safety and initial efficacy
NCT02416622	X-linked retinoschisis	rAAV-hRS1	RS1	rAAV2	Phase I/II	Ongoing, initial results promising

AMD, Age-related macular degeneration; DME, Diabetic macular edema; LCA, Leber congenital amaurosis.

While gene therapy offers significant promise, several challenges must be addressed. The immune response to viral vectors poses a risk of inflammation and could reduce the effectiveness of the therapy (18, 19). Additionally, the long-term efficacy of these therapies remains under investigation, as retinal degeneration is a complex process that may require combination therapies to achieve optimal outcomes (20). The high upfront cost of gene therapies, such as Luxturna (~$850,000 for both eyes), raises accessibility concerns, yet their potential to provide a one-time treatment could yield long-term pharmacoeconomic benefits by reducing ongoing care costs and vision loss-related expenses (e.g., caregiving, lost productivity) (21). A 2025 analysis from Precision Medicine Online suggests that newer therapies like Nanoscope’s retinal gene therapy may be more cost-effective, with estimated costs of $67,400–$101,300, as supported by the Institute for Clinical and Economic Review (ICER) (22). However, comprehensive pharmacoeconomic studies remain limited, and challenges like manufacturing scalability and long-term efficacy require further investigation to fully assess their economic viability. Researchers are also exploring ways to enhance the efficiency and safety of gene delivery, such as using non-viral methods like nanoparticles, which may reduce immunogenicity and improve targeting precision (23). Advances in gene editing technologies, including CRISPR-Cas9, offer the potential for more precise and durable genetic corrections (24). A key example is the CRISPR-gene editing system called EDIT-101. In a small Phase I/II study, 14 individuals with CEP290-associated LCA received an EDIT-101 injection in one eye and were followed for 3 years. Results indicated improved daytime and central vision for six participants, enhanced vision with corrective lenses for four, and an overall increase in vision-related quality of life for six, with no serious adverse events (25). The future holds the potential for significant advancements in gene therapy through the integration of cutting-edge technologies and personalized medicine, aimed at enhancing the precision, efficacy, and accessibility of these treatments.

## 3 Stem cell therapy

Stem cells are undifferentiated cells with the ability to develop into various specialized cell types, including RPE cells and photoreceptors. This is why stem cell therapy offers exciting potential for addressing degenerative ocular diseases, particularly for restoring vision by replacing damaged or degenerated retinal cells (Table 2) (26, 27). One example of this is the use of limbal stem cells (LSCs) in treating limbal stem cell deficiency (LSCD), a condition characterized by impaired or insufficient LSCs, leading to symptoms such as dryness, reduced vision, and photophobia (28). Since Kenyon and Tseng first developed limbal tissue transplantation in 1989 (29), a range of techniques has evolved. One such technique, cultivated limbal epithelium transplantation (CLET), uses autologous limbal cells and has shown significant long-term success and safety. For instance, autologous CLET has been reported to have better long-term survival and fewer complications than allogenic CLET, such as chronic inflammation and scarring (30). Other approaches include simple limbal epithelial transplantation (SLET), which simplifies the process by using a small biopsy of healthy limbal tissue for transplantation. Sangwan et al. demonstrated that SLET allows for the *in vivo* expansion of LSCs, reducing the need for donor tissue and making the procedure more cost-effective (31). Additionally, an ongoing clinical trial is comparing the efficacy of CLET and SLET in patients with LSCD caused by ocular burns, further evaluating which technique offers superior outcomes. SLET’s cost-effectiveness and reduced need for donor tissue make it a valuable alternative for treating LSCD in resource-limited settings (32). Moreover, advancements such as using contact lenses as a scaffold for limbal stem cell cultures have shown promise in delivering stem cells to damaged corneas efficiently, while also being more accessible and cost-effective (33). These developments represent significant progress in stem cell-based treatments for LSCD, offering hope for patients with severe ocular surface disorders.

**Table 2:** Clinical studies assessing effect of stem cell therapy in ophthalmology.

NCT ID	Condition	Study title	Phases	Outcomes
NCT01691261	AMD	A study of implantation of retinal pigment epithelium in subjects with acute wet AMD	Phase I	Ongoing, assessing safety and efficacy
NCT03102138	AMD	Retinal pigment epithelium safety study for patients in B4711001	Unknown	Not yet started
NCT04339764	AMD	Autologous transplantation of induced pluripotent stem cell-derived retinal pigment epithelium for geographic atrophy associated with AMD	Phase I/II	Ongoing, early results show promise
NCT05187104	AMD	Treatment of AMD using retinal stem and progenitor cells	Phase I/II	Early-phase, safety and preliminary efficacy data promising
NCT05991986	AMD	Preparation of patient autologous induced pluripotent stem cell-derived retinal cells for AMD	Unknown	Not yet started
NCT03981549	Central retinal vein occlusion	Treatment of central retinal vein occlusion using stem cells study	Phase I/II	Ongoing, some improvement in retinal health observed
NCT03990051	Chronic ocular GVHD	Treatment safety and efficacy of pro-ocular™ 1% for chronic ocular graft following allogeneic HSCT	Phase II	Completed, showed positive safety and efficacy
NCT04932629	Corneal scars and opacities	To evaluate the clinical safety and efficacy of limbal stem cell for treatment of superficial corneal pathologies	Early Phase I	Not yet started
NCT05705024	Corneal ulcer	Efficacy of locally delivered allogeneic mesenchymal stromal cells	Phase II	Ongoing, assessing safety and efficacy
NCT04627428	Dry AMD	Safety and tolerability of RPE stem cell-derived RPE (RPESC-RPE) transplantation in patients with dry AMD	Phase I/II	Ongoing, assessing safety and early efficacy
NCT04213248	DED	Effect of UMSCs derived exosomes on DED in patients with cGVHD	Phase I/II	Ongoing, early results show promise
NCT05738629	DED	Safety and efficacy of pluripotent stem cell-derived mesenchymal stem cell exosome (PSC-MSC-Exo) eye drops treatment for DEDs post refractive surgery and associated with blepharospasm	Phase I/II	Not yet started
NCT03302273	DED syndromes, DED, Ocular inflammation, Ocular surface disease, Ocular discomfort, Blepharitis	Corneal epithelial stem cells and DED	Unknown	Completed, assessing long-term outcomes
NCT03878628	DED, Kerato conjunctivitis sicca, Aqueous tear deficiency	Treatment with allogeneic adipose-derived mesenchymal stem cells in patients with aqueous deficient DED	Early Phase I	Completed, showed safety and some efficacy
NCT05147701	Eye diseases, Retinitis pigmentosa, Glaucoma, Diabetic retinopathy, Macular degeneration, Traumatic optic neuropathy, Optic atrophy	Safety of cultured allogeneic adult umbilical cord derived mesenchymal stem cells for eye diseases	Phase I	Ongoing, assessing safety and early efficacy
NCT05170347	GVHD, Hematological malignancy, Cancer	cGVHD after bone marrow transplantation: A territory-wide cohort	Unknown	Ongoing, large cohort study
NCT04792580	GVHD, Ocular GVHD	The effects and safety of 5% lifitegrast ophthalmic solution in subjects with DED in ocular GVHD	Early Phase I	Ongoing, assessing safety and efficacy
NCT04615455	Keratoconjunctivitis sicca, in sjogren’s syndrome	Mesenchymal stem cell therapy of DED in patients with sjogren’s syndrome	Phase II	Completed, positive safety and efficacy results
NCT04594512	Keratoconus, Keratoconus of right eye	Fresh corneal lenticule implantation and autologous serum - Case report	Unknown	Ongoing, case report
NCT04636918	Leukemia (Both ALL and AML), MDS-EB-1	Ikervis for DED Due to GVHD Post Allo-HSCT	Phase IV	Ongoing, assessing safety and efficacy
NCT03884569	LSCD	Cultivated limbal epithelial transplantation for LSCD	Unknown	Not yet started
NCT03957954	LSCD	Stem Cell therapy for LSCD	Phase I	Ongoing, assessing safety and early efficacy
NCT04995926	LSCD	Labial mucosal epithelium grafting for corneal limbus substitution	Unknown	Ongoing, assessing safety and early efficacy
NCT05909735	LSCD, Congenital aniridia	Treatment of LSCD with diabetes	Phase I	Ongoing, assessing safety and early efficacy
NCT04773431	Limbus corneae, Limbus corneae insufficiency syndrome	Safety evaluation of LSCD101 transplantation for LSCD	Phase I	Completed, safety established
NCT04642729	Macular corneal dystrophy	Fresh corneal lenticule implantation in macular corneal dystrophy with relax smile surgery	Unknown	Ongoing, assessing safety and efficacy
NCT05445063	Macular degeneration	Safety and efficacy of autologous transplantation of iPSC-RPE in the treatment of macular degeneration	Phase I	Not yet started
NCT05784519	Mesenchymal Stem Cell, DED Syndromes	Therapeutic effect of stem cell eye drops on DED	Early Phase I	Not yet started
NCT04877067	Methanol Poisoning, Toxic Optic Neuropathy, Stem Cell Tyrosine Kinase 1 Y842X, Magnetic Field Exposure	Therapy of toxic optic neuropathy via combination of stem cells with electromagnetic stimulation	Phase III	Completed, safety and efficacy data positive
NCT05658237	Myopic chorioretinal atrophy	Clinical study of PAL-222 targeting patients with myopic chorioretinal atrophy (PAMyCA)	Unknown	Ongoing, assessing safety and efficacy
NCT03829566	Neuromyelitis optica, Devic’s disease, NMO spectrum disorder	Autologous transplant to end NMO spectrum disorder	Phase II/III	Withdrawn, no outcomes available
NCT04552730	Neurotrophic keratitis	Nerve growth factor for the treatment of cornea disease	Unknown	Completed, safety and efficacy established
NCT05311514	Ocular GVHD	Allogeneic platelet lysate eye drops for the treatment of severe chronic ocular GVHD	Phase II	Ongoing, assessing safety and efficacy
NCT05279157	Ophthalmological disorder, Corneal dystrophy, Treatment, Therapy, Keratoconus	Autologous adipose-derived adult stem cell implantation for corneal diseases (ADASCs-CT-CD)	Phase II	Completed, safety and efficacy established
NCT06200727	Platelet-rich fibrin, Macular holes, pterygium, Glaucoma	Platelet-rich fibrin (PRF) membrane in ophthalmic diseases	Unknown	Ongoing, assessing safety and efficacy
NCT05528809	Primary Gougerot-Sjogren syndrome, Systemic sclerosis	Quantification and characterization of circulating epithelial and endothelial cells in Gougerot-Sjogren syndrome, compared to systemic sclerosis	Unknown	Ongoing, assessing safety and efficacy
NCT04490876	Proliferative vitreoretinopathy	Outcomes of extensive brilliant blue G-assisted internal limiting membrane peeling in proliferative vitreoretinopathy	Unknown	Completed, positive outcomes
NCT03944239	Retinitis pigmentosa	Safety and efficacy of subretinal transplantation of clinical human embryonic stem cell derived retinal pigment epitheliums in treatment of retinitis pigmentosa	Phase I	Not yet started
NCT04284293	Retinitis pigmentosa	CNS10-NPC for the treatment of retinitis pigmentosa	Phase I	Ongoing, assessing safety and early efficacy
NCT04604899	Retinitis pigmentosa	Safety of repeat intravitreal injection of human retinal progenitor cells (jCell) in adult subjects with retinitis pigmentosa	Phase II	Completed, safety and efficacy established
NCT04925687	Retinitis pigmentosa	Phase I study of intravitreal autologous CD34+ stem cell therapy for retinitis pigmentosa	Phase I	Ongoing, safety and early efficacy data positive
NCT05413148	Retinitis pigmentosa	The effect of stem cells and stem cell exosomes on visual functions in patients with retinitis pigmentosa	Phase II/III	Ongoing, assessing safety and efficacy

AMD, Age-related macular degeneration; DED, Dry eye disease; GVHD, Graft vs host disease; LSCD, Limbal stem-cell deficiency.

In retinal degenerative diseases, such as AMD and retinitis pigmentosa, stem cell-based therapies are advancing. One promising application is the replacement of RPE cells (34). RPE cells are essential for maintaining photoreceptors, and their degeneration leads to vision loss (35). Several studies have explored the transplantation of human embryonic stem cell (hESC)-derived RPE cells into the subretinal space. In a Phase I clinical study, patients with dry AMD showed improved visual acuity following the subretinal transplantation of hESC-derived RPE cells, with no signs of tumorigenicity or immune rejection over 4 months (36). In a separate study, a mismatched donor RPE monolayer implanted into a severely degenerated retina survived and functioned for 2 years, demonstrating limited immunogenicity of the allogeneic hESC-RPE cells (37). These early results suggest that RPE replacement can restore retinal function in AMD patients. Stargardt disease type 1 (STGD1), a hereditary form of macular degeneration, has also been a target for stem cell therapy. In a clinical trial involving the subretinal injection of hESC-derived RPE cell suspension, patients with early-stage STGD1 experienced no adverse reactions, although changes in visual function were variable (38). In another study using adipose-derived MSCs (ADMSCs) for suprachoroidal implantation, patients with STGD1 showed improvements in visual field and acuity. However, larger patient populations are needed to confirm the efficacy of this approach (39). In retinitis pigmentosa, characterized by the degeneration of rod and cone photoreceptors, early trials have focused on the safety and efficacy of transplanting retinal progenitor cells (RPCs). Patients with retinitis pigmentosa who received intravitreal injections of RPCs have shown improvements in visual acuity, although these gains were not sustained beyond 6 months (40). Alternatively, visual acuity score improvements for 50% of retinitis pigmentosa patients treated with neural RPC layers and RPE transplantation have been observed in another clinical trial (41). Additionally, neural precursor cell-derived astrocytes and RPE are being investigated for the treatment of retinitis pigmentosa (42), though extended research is needed to develop strategies that ensure long-term survival and integration of transplanted cells.

Glaucoma is another condition where stem cell therapy is being explored. The trabecular meshwork plays a crucial role in regulating intraocular pressure (IOP), and damage to trabecular meshwork cells leads to elevated IOP and optic nerve damage in glaucoma (43). Stem cell therapies, such as the injection of trabecular meshwork stem cells (TMSCs) into the anterior chamber, have shown potential in repopulating the trabecular meshwork and restoring its function. In studies using induced pluripotent stem cell-derived trabecular meshwork cells, both *ex vivo* and *in vivo* models demonstrated reduced IOP and restored TM function (44, 45). Notably, injecting mesenchymal stem cells (MSCs) directly into the ocular anterior chamber provides neuroprotection comparable to TMSC therapy. Based on a Phase I clinical study investigating trabecular meshwork regeneration (46), ADMSCs have been recommended for clinical trials due to their reduced risk of immune rejection and tumorigenesis (47). In a pilot study, intravenous injections of autologous bone marrow mesenchymal stem cells (ABMSCs) were administered to patients with diabetic retinopathy, a common complication of diabetes. Over 6 months, these patients showed reductions in macular thickness and improvements in visual acuity, suggesting that ABMSCs could offer a safe and effective treatment for diabetic retinopathy (48). Dry eye disease (DED), a multifactorial condition of the tear and ocular surface, is often associated with discomfort and vision impairment (49). Allogeneic ADMSCs injected into the lacrimal gland have been shown to reduce inflammation and improve symptoms in severe cases of DED. These stem cells can enhance tissue repair and modulate immune responses, providing relief to patients suffering from DED associated with conditions such as chronic graft-versus-host disease (cGVHD) (50). MSC-derived exosomes have also demonstrated efficacy in treating DED by alleviating inflammation and improving tear production when administered as eye drops. In addition, MSC-derived exosomes significantly reduced DED symptoms in patients with cGVHD (51), presenting a new approach for managing severe dry eyes.

Stem cell therapies, although promising, face challenges such as immune rejection, potential tumorigenicity, and the difficulty of ensuring long-term integration and function of transplanted cells (52, 53). However, ongoing developments in immune evasion strategies and the use of autologous stem cells may help mitigate some of these issues, enhancing the safety and efficacy of these therapies in the future (54). Pharmacoeconomic implications of stem cell therapies are also underexplored in current research. High initial costs for cell cultivation and delivery procedures may be offset by long-term savings if they halt disease progression and reduce the need for repeated interventions, potentially improving quality of life for patients with conditions like AMD (55). Studies suggest autologous approaches could lower costs, though scalability and regulatory hurdles pose economic challenges (36). Further pharmacoeconomic analyses are needed to quantify these benefits and guide clinical adoption.

## 4 Advanced imaging

**Figure 3:**
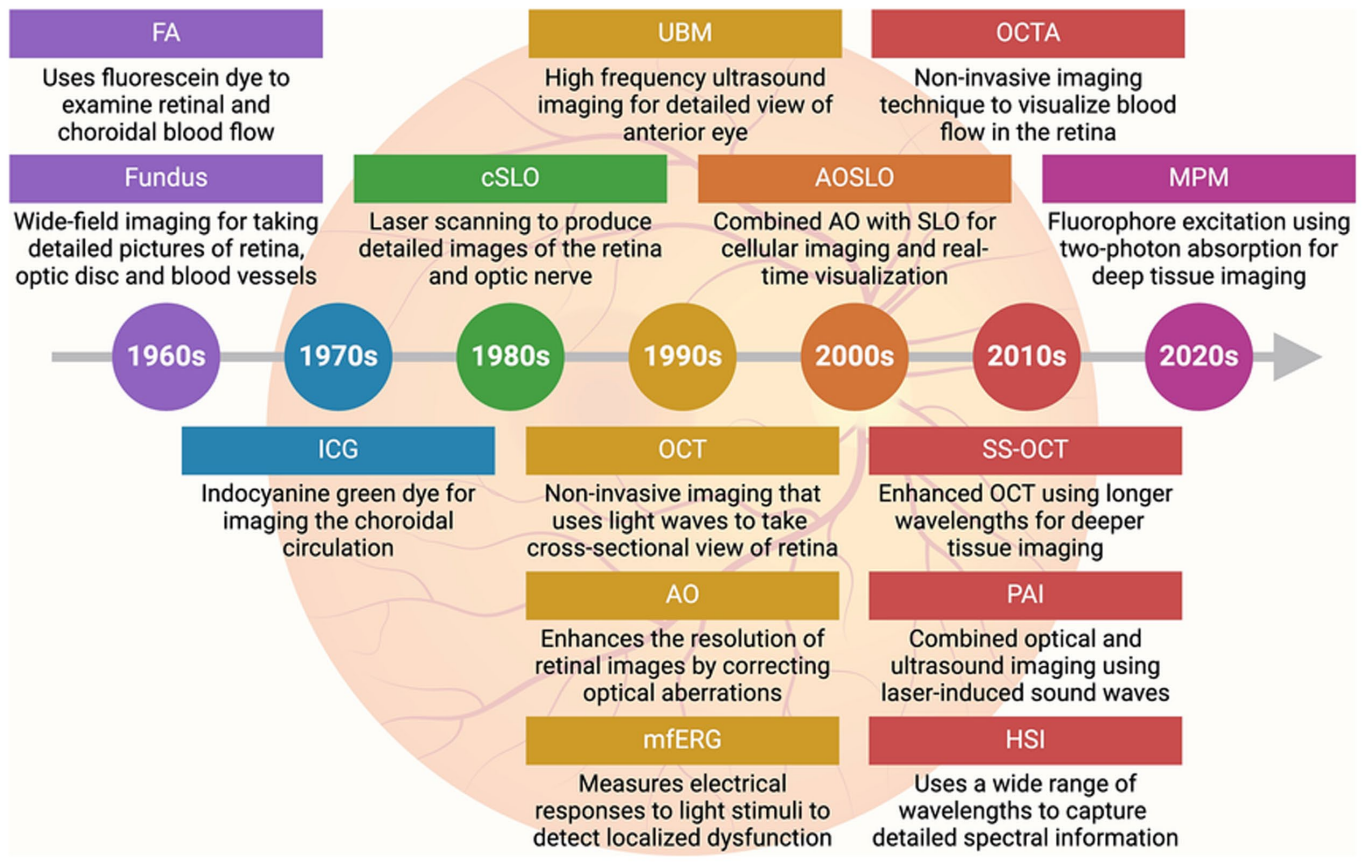
Advances in imaging technology in ophthalmology. Timeline (with brief description) of key imaging advancements made in the field of ophthalmology. FA, Fluorescein angiography; ICG, Indocyanine green angiography; cSLO, Confocal scanning laser ophthalmoscopy; UBM, Ultrasound biomicroscopy; OCT, Optical coherence tomography; AO, Adaptive optics; MfERG, Multifocal electroretinography; AOSLO, Adaptive optics scanning laser ophthalmoscopy; OCTA, Optical coherence tomography angiography; SS-OCT, Swept-source optical coherence tomography; PAI, Photoacoustic imaging; HSI, Hyperspectral imaging; MPM, Multiphoton microscopy.

Advanced imaging techniques have become indispensable in modern ophthalmology, offering critical insights into the structure and function of the retina and other ocular tissues (Figure 3 and Table 3) (56). Fluorescein angiography (FA), developed in the 1960s, was among the earliest techniques for visualizing retinal and choroidal blood flow. FA uses sodium fluorescein dye, which absorbs excitation light of wavelengths between 465 and 490 nm (blue) and emits light at wavelengths between 520 and 530 nm (yellow). A specialized camera captures detailed images of retinal blood vessels, enabling the detection of vascular abnormalities such as leakage and neovascularization. FA remains a valuable tool for diagnosing conditions like diabetic retinopathy and macular degeneration, providing essential insights into retinal vascular health (57, 58). Fundus photography, also emerging in the 1960s–1970s, captures detailed photographs of the retina, optic disc, and blood vessels. This technique is widely used for documenting and monitoring the progression of retinal and optic nerve diseases. Its ability to provide high-quality, wide-field images has made it a staple in ophthalmic diagnostics, facilitating long-term monitoring of conditions such as glaucoma and diabetic retinopathy (59). Indocyanine green angiography (ICG), introduced in the 1970s, provides an alternative to FA for visualizing the deeper choroidal circulation. ICG uses indocyanine green dye, which penetrates the RPE more effectively, making it particularly useful for diagnosing choroidal neovascularization and polypoidal choroidal vasculopathy. This technique offers a deeper view of the choroidal vasculature, providing insights into diseases affecting the deeper layers of the eye (60).

**Table 3:** Advanced imaging techniques in ophthalmology.

Imaging technique	Description	Applications	Advantages
Fluorescein angiography (FA) (1960s)	Uses fluorescein dye and a specialized camera to examine blood flow in the retina and choroid	Detection of retinal vascular disorders, diabetic retinopathy, and macular degeneration	Detailed view of retinal blood vessels, useful for identifying leakage
Fundus photography (1960s–1970s)	Imaging technique capturing detailed photographs of the retina, optic disc, and blood vessels	Documentation and monitoring of retinal and optic nerve diseases	Wide-field imaging, high-quality images
Indocyanine green angiography (ICG) (1970s)	Similar to FA but uses indocyanine green dye, better for imaging the choroidal circulation	Diagnosing choroidal neovascularization, polypoidal choroidal vasculopathy	Better penetration through retinal pigment, useful for choroidal imaging
Confocal scanning laser ophthalmoscopy (cSLO) (1980s)	Uses laser scanning to produce detailed images of the retina and optic nerve	Diagnosing and managing glaucoma, macular degeneration, and diabetic retinopathy	High-contrast images, ability to image specific retinal layers
Ultrasound biomicroscopy (UBM) (1990s)	High-frequency ultrasound imaging for detailed views of the anterior segment of the eye	Evaluating anterior segment structures, diagnosing glaucoma, and tumors	Detailed imaging of anterior segment structures, useful for opaque media
Optical coherence tomography (OCT) (1990s)	Non-invasive imaging test that uses light waves to take cross-sectional pictures of the retina	Diagnosis and management of retinal diseases like macular degeneration, diabetic retinopathy, and glaucoma	High-resolution images, real-time imaging, and early disease detection
Adaptive optics (AO) (1990s)	Enhances the resolution of retinal images by correcting optical aberrations	Detailed imaging of photoreceptors, early detection of retinal diseases	Ultra-high resolution images, real-time correction of optical distortions
Multifocal electroretinography (mfERG) (1990s)	Measures electrical responses of various retinal areas to light stimuli	Assessing localized retinal function, diagnosing retinal disorders	Provides functional assessment of the retina, useful for detecting localized dysfunction
Adaptive optics scanning laser ophthalmoscopy (AOSLO) (2000s)	Combines adaptive optics with scanning laser ophthalmoscopy for high-resolution imaging of the retina	Imaging individual photoreceptors, retinal blood flow, and detailed retinal structure	Extremely high-resolution images, allows for cellular-level imaging, real-time visualization
Optical coherence tomography angiography (OCTA) (2010s)	Non-invasive imaging technique to visualize blood flow in the retina	Detecting and monitoring retinal vascular diseases, diabetic retinopathy	Non-invasive, detailed visualization of blood flow, no dye required
Swept-source optical coherence tomography (SS-OCT) (2010s)	Enhanced version of OCT using longer wavelengths for deeper tissue imaging	Imaging deeper retinal and choroidal structures, diagnosing retinal diseases	Faster image acquisition, deeper penetration, improved image quality
Photoacoustic imaging (PAI) (2010s)	Combines optical and ultrasound imaging using laser-induced sound waves	Imaging oxygen saturation, blood vessel networks, early detection of tumors and vascular abnormalities	Non-invasive, provides functional data like oxygen levels, deep tissue imaging
Hyperspectral imaging (HSI) (2010s–2020s)	Uses a wide range of wavelengths to capture detailed spectral information	Detection of early changes in diabetic retinopathy, age-related macular degeneration	Provides metabolic and functional data in addition to structural imaging
Multiphoton microscopy (MPM) (2020s)	Advanced microscopy technique that excites fluorophores using two-photon absorption	Detailed imaging of retinal cells, tracking cellular activity in vivo	High-resolution, deep tissue imaging, minimal damage to tissues

Confocal scanning laser ophthalmoscopy (cSLO), developed in the 1980s, uses laser scanning to produce high-contrast images of the retina and optic nerve. It is highly effective for diagnosing and managing glaucoma, macular degeneration, and diabetic retinopathy (61). Recent methods have enabled real-time visualization of retinal ganglion cells (RGCs) using cSLO, allowing for longitudinal studies to monitor RGC survival and disease progression or remission following treatment (62). Ultrasound biomicroscopy (UBM), introduced in the 1990s, provides high-frequency ultrasound imaging of the anterior segment of the eye, including the iris, ciliary body, and anterior chamber angle. UBM has proven useful in diagnosing and managing glaucoma and anterior segment tumors (63). Advancements toward three-dimensional (3D) UBM have enabled detailed visualization and measurement of anterior eye tissues in a 3D context, aiding in treatment planning such as lens placement and microcatheter cannulation (64). Optical coherence tomography (OCT), a major breakthrough of the 1990s, revolutionized ophthalmic imaging by using light waves to capture high-resolution, cross-sectional images of the retina. OCT allows clinicians to visualize retinal layers in great detail, making it a cornerstone in diagnosing and managing AMD, diabetic retinopathy, and glaucoma. By detecting subtle changes in retinal thickness and other pathological features, OCT facilitates early diagnosis and intervention, crucial for preserving vision (65). Multifocal electroretinography (mfERG), also developed in the 1990s, measures the electrical responses of different areas of the retina to light stimuli, providing a functional assessment of localized retinal regions. Unlike traditional ERG, mfERG can detect localized dysfunction, making it especially useful for diagnosing and monitoring early diabetic retinopathy and retinitis pigmentosa (66).

Adaptive optics (AO), introduced in the 1990s, has been instrumental in enhancing retinal imaging by correcting optical aberrations caused by the eye’s optics. AO enables detailed imaging of photoreceptors and other fine retinal structures, providing new insights into the pathophysiology of retinal diseases (67). The combination of AO with scanning laser ophthalmoscopy (AOSLO), developed in the 2000s, further enhanced the resolution and contrast of retinal images. AOSLO allows real-time visualization of cellular structures in the retina, which is particularly valuable for monitoring inherited retinal diseases, such as retinitis pigmentosa (68). This technique is also used to assess the efficacy of gene and stem cell therapies at the cellular level (69). Optical coherence tomography angiography (OCTA), developed in the 2010s, expanded OCT capabilities by allowing visualization of retinal and choroidal blood flow without dye injection. OCTA provides detailed maps of the retinal vasculature, making it useful for detecting ischemia, neovascularization, and vascular abnormalities in conditions like AMD and diabetic retinopathy. It also enables non-invasive monitoring of treatment efficacy and disease progression (70). Swept-source optical coherence tomography (SS-OCT), introduced in the 2010s, is an advanced form of OCT that uses longer wavelengths for deeper tissue imaging. SS-OCT allows faster image acquisition and better visualization of the choroid and deeper retinal structures, making it highly valuable for diagnosing diseases affecting these areas (71). Photoacoustic imaging, also developed in the 2010s, combines optical and ultrasound imaging using laser-induced sound waves. This technique provides insights into oxygen saturation and blood vessel networks in the retina, aiding in the early detection of tumors and vascular abnormalities (72, 73). Hyperspectral imaging, emerging in the 2010s and continuing into the 2020s, uses a wide range of wavelengths to capture detailed spectral information about ocular tissues. This technique has been particularly useful in detecting early changes in diabetic retinopathy and AMD, as it provides metabolic and functional data alongside structural imaging (74). Finally, multiphoton microscopy, gaining traction in the 2020s, is an advanced microscopy technique that excites fluorophores using two-photon absorption. It allows high-resolution, deep tissue imaging, which is useful for tracking cellular activity *in vivo* (75, 76). This technique has opened new possibilities for understanding the cellular mechanisms underlying retinal diseases and their response to treatments (77). As these technologies continue to evolve, they hold the potential to enhance early diagnosis, guide personalized treatments, and improve long-term outcomes for patients with ocular diseases.

## 5 Novel therapeutics

Therapeutic advancements are making significant strides in improving the treatment of various ocular diseases. Among these, anti-angiogenesis, anti-fibrosis, and neuroprotection stand out as major areas of focus (Table 4) (78, 79). Anti-VEGF drugs have revolutionized the treatment of neovascular AMD and diabetic macular edema (DME) (79). These conditions, characterized by abnormal blood vessel growth and fluid leakage in the retina, can lead to severe vision loss if left untreated. Anti-VEGF therapies work by inhibiting VEGF, a protein that promotes the growth of these abnormal vessels. By blocking VEGF, these drugs help reduce fluid accumulation, stabilize the retina, and prevent further vision deterioration (80, 81). The most widely used anti-VEGF drugs include ranibizumab (Lucentis) and aflibercept (Eylea), both of which have demonstrated efficacy in numerous clinical trials (82–84). However, high per-dose costs (e.g., $1,000–$2,000 per injection) and the frequent dosing schedules required for these treatments—often involving monthly injections—pose a significant burden for patients and healthcare systems (82–84). This has driven the development of newer formulations and delivery methods, such as extended-release options, to reduce the frequency of injections while maintaining or improving treatment efficacy in AMD (85, 86). Brolucizumab (Beovu) is a newer anti-VEGF drug that has garnered attention for its longer duration of action compared to earlier treatments. Approved by the FDA in 2019 for the treatment of neovascular AMD, brolucizumab offers the advantage of less frequent dosing, potentially reducing the treatment burden for patients (87). Clinical trials, including the HAWK and HARRIER studies, have demonstrated that brolucizumab is effective in reducing retinal fluid and improving vision in patients with neovascular AMD, with some patients able to extend their treatment intervals to 12 weeks or more (88). These findings suggest that brolucizumab could offer a more convenient treatment option for patients, particularly those who struggle with the logistics and discomfort of frequent injections. Overall, these therapies may justify their expense by delaying vision loss, yet specific cost-effectiveness data is lacking (89). Future research should focus on quantifying long-term economic benefits to optimize their clinical use.

**Table 4:** Clinical studies assessing effect of novel therapeutics in ophthalmology.

NCT ID	Condition	Drug	Mechanism of action	Delivery method	Phase	Primary endpoint
NCT03211234	Neovascular AMD	Ranibizumab (Lucentis)	Anti-VEGF	Intravitreal injection	Phase IV	Long-term safety, visual acuity maintenance
NCT02660524	Neovascular AMD	Aflibercept (Eylea)	Anti-VEGF	Intravitreal injection	Phase IV	Visual acuity, safety
NCT02307682	Neovascular AMD	Brolucizumab (Beovu)	Anti-VEGF	Intravitreal injection	Phase III	Reduction in retinal fluid, visual acuity
NCT04226934	Neovascular AMD	Faricimab	Anti-VEGF/Anti-Ang2 dual inhibition	Intravitreal injection	Phase III	Visual acuity, retinal fluid reduction
NCT03706179	DME	Abicipar Pegol	Anti-VEGF	Intravitreal injection	Phase III	Reduction in retinal thickness, visual acuity
NCT04065490	DME	Conbercept	Anti-VEGF	Intravitreal injection	Phase III	Reduction in macular edema, improvement in visual acuity
BETTER Trial	AMD, DME	ISTH0036	Anti-TGF-β	Intravitreal injection	Phase II	Reduction in retinal thickness, Stable IOP, improved visual acuity
NCT03889652	Glaucoma	Brimonidine	Neuroprotection, IOP lowering	Topical ophthalmic solution	Phase II	Preservation of RGCs, progression of visual field loss
NCT02501156	Glaucoma	Latanoprostene Bunod	IOP lowering, neuroprotection	Topical ophthalmic solution	Phase III	Reduction in IOP, visual field preservation
NCT04641464	Glaucoma	Cytidine-5-diphosphocholine (CDP-Choline)	Neuroprotection	Oral administration	Phase II	Preservation of RGCs, progression of visual field loss
NCT02749734	Glaucoma	Trabodenoson	Neuroprotection, IOP lowering	Topical ophthalmic solution	Phase II/III	Reduction in IOP, preservation of visual field

AMD, Age-related macular degeneration; DME, Diabetic macular edema.

Targeting transforming growth factor-beta (TGF-β) for ocular conditions has also gained attention in recent years due to its involvement in fibrosis and abnormal vascularization in the retina (90). In this context, ISTH0036, an antisense oligonucleotide targeting TGF-β2, is progressing in trials for treating retinal diseases. In a Phase I trial involving glaucoma patients undergoing trabeculectomy, intravitreal ISTH0036 was well-tolerated with no related adverse events and effectively maintained IOP below 10 mmHg (91). In an ongoing Phase II study, ISTH0036 has shown potential in reducing fibrosis and fluid volumes in treatment-naïve and anti-VEGF-pretreated patients with wet AMD and DME. The treatment led to improved best-corrected visual acuity and reduced central retinal thickness, with stable IOP and minimal cataract worsening (92). These results highlight ISTH0036’s promise as a novel anti-fibrotic therapy for retinal conditions.

Neuroprotective agents represent a promising approach for treating neurodegenerative eye diseases, such as glaucoma and optic neuropathies (93). Glaucoma is characterized by the progressive degeneration of RGCs and their axons, which form the optic nerve. Current treatments for glaucoma primarily focus on lowering IOP, the main modifiable risk factor for the disease (94). However, lowering IOP alone does not prevent disease progression in all patients, particularly those with normal-tension glaucoma or advanced disease. Consequently, there is growing interest in developing therapies that can protect RGCs from degeneration, offering additional benefits beyond conventional IOP-lowering treatments (95). One of the most studied neuroprotective agents in glaucoma is brimonidine, an alpha-2 adrenergic agonist traditionally used as an IOP-lowering agent (96). Brimonidine has been shown to have neuroprotective effects in preclinical models, where it promotes the survival of RGCs and reduces axonal damage in the optic nerve (97). Its mechanism of action is believed to involve the upregulation of anti-apoptotic pathways, inhibition of excitotoxicity, and reduction of oxidative stress within the retina (98–100). Early clinical studies in glaucoma patients suggest that brimonidine may help preserve RGCs and slow the progression of the disease, making it a promising candidate for neuroprotection in glaucoma (101). As research in this area continues, identifying additional neuroprotective targets and optimizing drug delivery to the retina will be crucial for translating these promising agents into effective clinical therapies. The ultimate goal of these advancements is to provide more effective, convenient, and long-lasting treatments that can improve patient outcomes and quality of life.

## 6 Nanotechnology

Nanotechnology is rapidly emerging as a transformative field in ophthalmology, offering innovative solutions for drug delivery, imaging, and tissue engineering (6). Among its most promising applications is drug delivery. Traditional methods often face challenges such as poor bioavailability, rapid degradation, and limited penetration into ocular tissues (102). Nanoparticles can overcome these issues by providing controlled and sustained release of therapeutic agents, improving drug stability, and enhancing tissue penetration (103). For example, cyclodextrin-based nanoparticles have been developed to deliver dexamethasone for treating DME. These nanoparticles enhance the solubility and bioavailability of dexamethasone, enabling effective delivery to the retina. Clinical trials have demonstrated that nanoparticle-based dexamethasone formulations significantly reduce macular thickness and improve visual acuity in patients with DME (104–106), presenting a potential alternative to traditional steroid injections. In glaucoma, nanoparticles are being developed to enhance drug delivery for reducing IOP. For example, liposomal nanoparticles carrying latanoprost have been studied for their ability to provide sustained drug release, reducing administration frequency and improving patient compliance (107). Early clinical trials suggest these nanoparticles effectively lower IOP, potentially offering an improvement over traditional eye drops (108). Nanotechnology also holds promise for non-invasive treatments in ophthalmology. Topical nanoparticle formulations are being investigated for conditions such as cataracts and dry eye syndrome (109). For cataract treatment, polymeric nanoparticles carrying urea have shown potential in dissolving cataractous lens opacities (110). Although still in early research stages, this approach could offer a non-surgical alternative to cataract removal. For dry eye syndrome, liposomal nanoparticles have been developed to deliver artificial tears and anti-inflammatory agents, enhancing the retention time of therapeutic agents on the ocular surface and providing longer-lasting relief from dry eye symptoms (111). A comprehensive list of clinical studies evaluating different nano-formulated drugs in ophthalmology is provided in Table 5.

**Table 5:** Clinical studies assessing effect of nanotechnology-based therapeutics in ophthalmology.

NCT ID	Condition	Nanoparticle	Drug carried	Delivery method	Phase	Outcome
NCT05105607	AMD	D-4517.2 (Hydroxyl Dendrimer)	VEGFR Tyrosine Kinase Inhibitor	Subcutaneous injection	Phase I	Early results suggest good safety profile; efficacy to be determined
NCT03249740	Neovascular AMD	Sunitinib Malate (GB-102) MP	Aflibercept	Intravitreal injection(s)	Phase I	Safe administration; efficacy under investigation
NCT03835884	Neovascular AMD	AR-13503 implant	Aflibercept	Intravitreal implant	Phase I	Safe administration observed; efficacy evaluation ongoing
NCT03001466	Cataracts	Pluronic® F-127(PF) polymeric NP	Urea	Topical	Phase II	Preliminary results indicate some improvement in lens clarity
NCT04130802	Corneal inflammation and post-operative pain	OCS-01 (Cyclodextrin NP)	Dexamethasone	Topical	Phase II	Significant reduction in inflammation and pain reported
NCT01523314	DME	Cyclodextrin NP	Dexamethasone	Topical	Phase II/III	Ongoing; preliminary results show significant reduction in macular thickness and improvement in vision
NCT03598699	DED	AXR-159 ophthalmic solution (Micelles)	Integrins α4β1 and α4β7 antagonists	Topical	Phase II	Demonstrates potential efficacy in reducing DED symptoms
NCT02908282	DED	REMOGEN® OMEGA (Microemulsion of polyunsaturated fatty acids and hydrating polymers)	Omega-3 fatty acids	Topical	Not applicable	Improvement in DED symptoms reported by participants
NCT02420834	DED	Liposomes	Artificial tears	Spray	Not applicable	Positive feedback on symptom relief and patient comfort
NCT03140111	DED secondary to Sjögren Syndrome	(LAMELLEYE) Liposomal NP	Slecithin phospholipids, sphingomyelin and cholesterol, suspended in saline	Topical	Not applicable	Early results suggest symptom improvement in DED severity
NCT02813265	DED, keratoconjunctivitis sicca	KPI-121 (submicron suspension)	loteprednol etabonate	Topical	Phase III	Positive results showing reduced symptoms and improved comfort
NCT01987323	Glaucoma	EggPC liposomes	Latanoprost	Subconjunctival injection	Phase I/II	Early results promising; decrease in intraocular pressure observed
NCT02371746	Glaucoma	ENV 515	Travoprost	Intracameral implant	Phase II	Promising results with sustained reduction in intraocular pressure
NCT00738361	Intraocular melanoma	Albumin-stabilized nanoparticle	Paclitaxel	Intravenous injections	Phase II	Completed; some patients show tumor size reduction
NCT03739593	Macular edema due to retinal vein occlusion	AR-1105	Dexamethasone	Intravitreal implant	Phase II	Interim results indicate effective reduction in macular edema
NCT03093701	Macular edema; Retinal vein occlusion	TLC399 (ProDex) Multi-layered lipid NP	Dexamethasone	One-time intravitreal injection	Phase II	Interim analysis indicates reduction in macular edema and improved visual acuity
NCT03617315	Meibomian gland dysfunction	Ethylenediaminetetraacetic acid (EDTA) disodium salt and crocin liposomes	Hyaluronic acid	Topical	Not applicable	Study ongoing; initial feedback suggests improvement in gland function
NCT03785340	Meibomian gland dysfunction	Nanoemulsion (OCU-310)	Brimonidine Tartrate	Topical	Phase III	Positive results with significant symptom relief and gland function improvement
NCT02163824	Ocular infections, irritations, and inflammation	KPI-121 (submicron suspension)	loteprednol etabonate	Topical	Phase III	Positive results with significant reduction in inflammation and symptom relief
NCT04008771	Retinitis Pigmentosa	SeeQ CdSe 655 Alt Nanoparticles (cadmium-selenium) NP	SeeQ Device	Two intravitreal injections	Phase I	Safety established; efficacy endpoints yet to be met

AMD, Age-related macular degeneration; DME, Diabetic macular edema; DED, Dry eye disease.

In addition to nano-medicines undergoing clinical evaluation, several nanoformulations for ocular therapies have been developed and commercialized. Drug-free nanoemulsions, such as Restasis®—the first nanoemulsion-based product containing cyclosporin A for chronic dry eye treatment—have been approved (112). Durezol®, another nanoemulsion containing difluprednate, is approved for eye inflammation treatment. Additionally, Visudyne®, a liposomal formulation of verteporfin marketed by Novartis Pharma AG, received FDA approval in 2000 for intravenous treatment of choroidal neovascularization associated with conditions like AMD, pathological myopia, and ocular histoplasmosis syndrome (113, 114). Macugen®, a PEGylated anti-VEGF aptamer, was approved by the FDA in 2004 for treating wet AMD via intravitreal injection (115). As of 2024, SYSTANE®, a propylene glycol-based nanoemulsion for dry eye treatment, has completed Phase IV clinical trials and is commercially available (116, 117). The increasing number of nano-based ocular therapies in clinical trials and on the market highlights the promise of nanotechnology in ophthalmology. However, further research is needed to optimize nanostructure delivery to the eye, address safety and biocompatibility concerns, and understand the long-term effects of nanoparticle accumulation in ocular tissues (118, 119). Nanotechnology’s potential to enhance drug delivery offers pharmacoeconomic promise by improving bioavailability and reducing treatment frequency, though specific cost data is absent from current studies. Initial development costs may be high, but sustained-release nanoparticles could lower long-term expenses by minimizing drug waste and injections, as seen in trials for DME (104–106). Nano-based systems may be cost-effective for chronic conditions, yet manufacturing scalability and safety concerns require further economic evaluation (6, 120). More studies are needed to validate these benefits. Additionally, challenges related to the manufacturing and scalability of nanoparticle-based therapies need to be addressed for widespread clinical adoption (121).

## 7 Artificial intelligence

**Figure 4:**
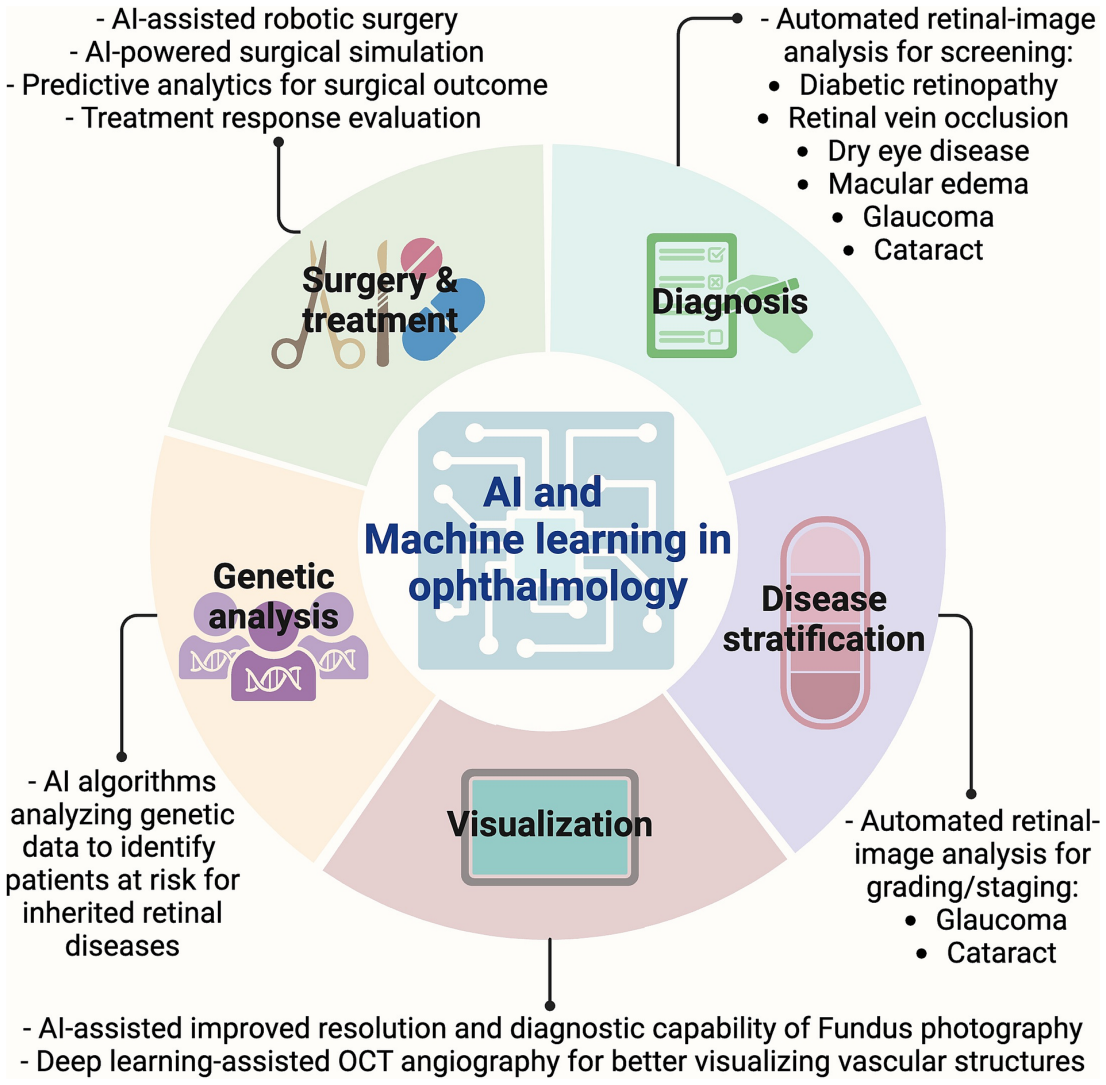
AI and machine learning in ophthalmology. Graphic showing timeline of key imaging advancements made in the field of ophthalmology. AI, Artificial intelligence; OCT, Optical coherence tomography.

Since its inception in 1956, AI has made significant strides in medical science, optimizing efficiency and driving technological innovations. AI’s transformative impact extends across various medical specialties, including ophthalmology, where it has facilitated improvements in patient care through enhanced data analysis for diagnosis and disease stratification, improved imaging and visualization, automated genetic analysis, and aid in surgical procedures (Figure 4 and Table 6) (122, 123). One of the most impactful applications of AI in ophthalmology is the screening and diagnosis of diabetic retinopathy, a leading cause of vision loss worldwide. The use of convolutional neural networks (CNNs) has been pivotal in this regard. Initial studies in 2016 demonstrated that CNNs could accurately detect diabetic retinopathy, achieving high areas under the curve (AUC) values of 0.980 and 0.991 (124, 125). Subsequent research, utilizing deep learning systems, further validated these findings in larger datasets, achieving an AUC of 0.93 with 90.5% sensitivity and 91.6% specificity for referable diabetic retinopathy, and an AUC of 0.958 with 100% sensitivity and 91.1% specificity for vision-threatening diabetic retinopathy (126). Today, AI algorithms can analyze retinal images to detect early signs of diabetic retinopathy, such as microaneurysms, hemorrhages, and exudates, with accuracy comparable to that of expert ophthalmologists. For instance, the EyeArt v2.1 system detects referable diabetic retinopathy with 95.7% accuracy (127). Beyond standard fundus photography, AI applications have extended to OCT and ultra-widefield imaging, which enhance the detection of diabetic-related peripheral diseases and macular edema. For example, a CNN model developed on OCT images detected macular edema with a cross-validation Dice coefficient of 0.911 (128), while a CNN developed on ultra-widefield images identified proliferative diabetic retinopathy with 94.7% sensitivity and 97.2% specificity (129).

**Table 6:** Advances in AI and machine learning in ophthalmology.

Application	Description	Impact	Year
Automated retinal image analysis	Machine learning algorithms automatically analyze retinal images for various pathologies.	Streamlined workflow for ophthalmologists, improving efficiency and accuracy.	2015
Diabetic retinopathy screening	AI algorithms analyze retinal images to detect signs of diabetic retinopathy.	Enhanced early detection and treatment, reducing vision loss from diabetes.	2016
Cataract detection and grading	AI applications evaluate lens opacity in images to detect and grade cataracts.	Improved preoperative assessment and postoperative monitoring of cataract surgery.	2016
AMD staging	AI systems evaluate retinal scans to identify and classify different stages of AMD.	Facilitated early diagnosis and better monitoring of disease progression.	2017
Glaucoma detection	Machine learning models assess optic nerve images and visual field tests to diagnose glaucoma.	Improved accuracy and efficiency in glaucoma diagnosis, enabling timely intervention.	2018
OCT analysis	AI interprets OCT images to diagnose and monitor retinal diseases such as macular edema and retinal detachment.	Enhanced interpretation of complex OCT data, aiding in precise diagnosis and treatment planning.	2018
Retinal vein occlusion detection	AI tools analyze fundus photographs to detect retinal vein occlusion.	Faster and more accurate identification of retinal vein occlusions, leading to prompt treatment.	2019
Predictive analytics for surgical outcomes	Machine learning models predict outcomes of ophthalmic surgeries based on patient data and surgical parameters.	Personalized surgical planning and improved patient outcomes.	2019
DED detection	AI algorithms analyze ocular surface images and tear film parameters to diagnose DED.	Enhanced accuracy and early detection of DED, leading to better management and treatment.	2020
AI-assisted robotic surgery	Integration of AI with robotic systems to assist in precise and minimally invasive eye surgeries.	Increased precision and reduced recovery time for patients undergoing eye surgeries.	2021
AI-enhanced fundus photography	Advanced AI models improve the resolution and diagnostic capability of fundus photography.	More accurate and earlier detection of retinal diseases, improving patient outcomes.	2022
Deep learning for OCT angiography	Deep learning techniques applied to OCT angiography for better visualization and diagnosis of vascular structures in the retina.	Enhanced detection and monitoring of retinal vascular diseases, leading to better patient care.	2022
Real-time AI diagnostic tools	AI-driven tools providing real-time analysis and diagnosis during eye examinations.	Immediate insights for ophthalmologists, improving decision-making and patient care.	2023
AI-driven genetic analysis	AI algorithms analyzing genetic data to identify patients at risk for inherited retinal diseases.	Early intervention and personalized treatment plans for patients with genetic predispositions.	2023
AI-powered surgical simulation	AI-based simulation tools for training ophthalmic surgeons, enhancing surgical skills through virtual reality and machine learning.	Improved training outcomes and surgical precision, reducing the risk of complications.	2024

AMD, Age-related macular degeneration; AI, Artificial intelligence; DED, Dry eye disease; OCT, Optical coherence tomography.

AMD treatment also benefits from AI’s ability to analyze large datasets of OCT and retinal photographs. A deep learning algorithm developed from a database of over 130,000 images from 4,613 patients achieved 92% accuracy in detecting moderate to advanced AMD (130). An independent study demonstrated that combining deep learning modalities, such as fundus photographs, OCT, and OCT angiography scans, could increase detection accuracy for AMD to 96% (131). AI also plays a role in quantifying AMD features such as intraretinal fluid and subretinal hyperreflective material, with recent advancements allowing for precise monitoring of treatment responses (132, 133). AI’s potential extends beyond diagnosing specific conditions to encompass broader retinal health assessments. An OCT-based deep learning system achieved a 99.21% AUC in identifying various retinal diseases and demonstrated proficiency in detecting conditions such as neovascular AMD and macular edema, showing comparable performance to that of retinal specialists (134). Glaucoma, the second leading cause of irreversible blindness, also benefits from AI innovations. Machine learning models can analyze optic nerve images and visual field tests to detect glaucoma at an early stage, when treatment is most effective. These models can also predict disease progression and identify patients at high risk of rapid vision loss, allowing for more personalized treatment strategies (135). For instance, deep learning systems have accurately differentiated glaucomatous damage from healthy eyes, with AUCs of 0.944 and 0.940 (136). These findings have been replicated through independent studies worldwide (137–139). Additionally, a deep learning-based CNN system trained on OCT images has efficiently differentiated progressing from non-progressing glaucoma (140). Notably, deep learning networks have been developed to predict the development of glaucomatous visual field changes up to 5 years into the future with high accuracy (141).

AI is also aiding in cataract detection, grading, and surgical assistance. The DeepLensNet, a deep-learning model designed to detect and quantify cataracts from slit-lamp and retroillumination images, has been introduced. This model accurately grades nuclear sclerosis, cortical lens opacity, and posterior subcapsular cataracts, showcasing its potential for automating cataract evaluation and improving global accessibility (142). Additionally, ResNet50 and XGBoost classifiers screen for visually significant cataracts from fundus images, achieving AUCs of 0.916–0.965. This approach streamlines the screening process using a single imaging modality, simplifying cataract detection and allowing integration with existing AI systems for posterior-segment diseases (143). Furthermore, an AI-driven platform has been developed to enhance phacoemulsification cataract surgery, providing real-time guidance to surgeons and achieving high accuracy in phase recognition and pupil segmentation. Surgeons have found this system useful, suggesting that AI could play a transformative role in both cataract screening and surgery (144). Meanwhile, AI also has made a significant breakthrough in areas of refractive surgery, cornea, and optic nerve diseases.

Despite the tremendous potential of AI in ophthalmology, several challenges must be addressed. Ensuring the accuracy and reliability of AI algorithms requires large, diverse datasets and rigorous validation (122, 145). Additionally, integrating AI into clinical practice requires careful consideration of ethical and legal issues, including patient privacy and data security. The reliance on vast amounts of patient data to train AI models underscores the critical need for robust data privacy measures, informed consent processes, and cybersecurity protocols to protect sensitive information. In this context, a smartphone-based offline AI system with high sensitivity for detecting diabetic retinopathy has been developed (146). The risk of algorithmic bias is also a pressing concern, as models trained on non-representative datasets may inadvertently perpetuate healthcare disparities, making it essential to develop transparent and explainable AI systems that can be scrutinized for fairness and accuracy (147, 148). Accountability in AI-driven clinical decision-making further complicates the landscape, as the diffusion of responsibility among developers, healthcare providers, and institutions calls for clearly defined ethical guidelines and regulatory frameworks to delineate liability in cases of diagnostic or treatment errors (149). Societally, while AI holds the promise of extending high-quality ophthalmic care, particularly in underserved regions, its integration also necessitates a transformation in workforce dynamics, with healthcare professionals requiring specialized training to effectively interpret and manage AI outputs (122, 150). Cost-effectiveness analysis of integration of AI into clinical practice is also necessary, though has not fully explored. By automating screening and reducing specialist reliance, AI tools like IDx-DR could lower costs, particularly in underserved areas, while early intervention may prevent expensive vision loss outcomes (151). AI-driven screening is also cost-effective, with savings from reduced referrals, though initial development costs are significant (152, 153). Ongoing research should further delineate these economic advantages to support broader implementation. Finally, the evolving regulatory environment, marked by efforts from bodies such as the FDA and the European Commission, is pivotal in setting rigorous safety and efficacy standards for AI applications in healthcare, thus ensuring that innovation is balanced with patient protection and ethical responsibility.

## 8 Teleophthalmology

Teleophthalmology, the application of telemedicine in ophthalmic care, has experienced rapid growth in recent years, particularly during the COVID-19 pandemic. This approach utilizes digital technology to provide remote eye care services, expanding access to diagnosis, monitoring, and treatment for patients who may not have easy access to specialized care (154). One of the major breakthroughs has been the use of teleophthalmology for screening retinal diseases. Diabetic retinopathy screening has been one of the most successful implementations of telemedicine, especially in rural and underserved communities where access to ophthalmologists is limited. Teleophthalmology programs, such as those developed in India, have established efficient workflows for capturing retinal images using portable devices and transferring them for remote diagnosis by specialists. This model has been shown to reduce unnecessary referrals and facilitate timely treatment, which is crucial for preventing vision loss (155). Similarly, tele-glaucoma monitoring has made significant strides with devices like the iCare HOME tonometer. Studies have demonstrated that this device, combined with AI algorithms, facilitates continuous monitoring of IOP and optic nerve health, allowing for better management of glaucoma by providing timely data to guide treatment decisions (156). Smart contact lenses with embedded sensors, anticipated in 2024, will enable continuous monitoring of IOP and facilitate timely interventions for glaucoma patients (157). A breakthrough phase 3 randomized controlled trial, involving 105 children aged 4–7 years with amblyopia, has evaluated dichoptic digital therapeutic against full-time glasses wear alone. Participants in the treatment group used the therapeutic at home for 1 h per day, 6 days a week, while the control group continued with glasses only. At 12 weeks, the amblyopic eye visual acuity improved by 1.8 lines in the treatment group compared to 0.8 lines in the control group, a difference that reached statistical significance and led to early study termination for success. Importantly, no serious adverse events were reported, supporting the therapeutic’s safety and clinical efficacy as an alternative treatment for amblyopia (158). However, a recent systematic review evaluating home-based screening tools for amblyopia in children, analyzed data from 28 studies involving various platforms such as smartphone/tablet applications, internet-based tools, digital cameras, and visual acuity charts. The review found significant variability in diagnostic accuracy, and noted methodological limitations, including selection biases and inconsistent recruitment methods. These issues highlight the need for standardized protocols and higher-quality studies to validate the efficacy of these home-based screening tools for amblyopia (159). Remote postoperative care is another important application of teleophthalmology, allowing patients recovering from eye surgeries to receive follow-up care through telemedicine platforms (160). This approach improves patient outcomes and convenience by reducing the need for frequent in-person visits and enabling closer monitoring of recovery progress.

The role of AI in teleophthalmology represents another key advancement, particularly in the development of deep learning systems for automated diagnosis (161). A notable development is the use of AI-powered platforms for diabetic retinopathy screening. The National Health Service in the UK has implemented a widely recognized AI integration program for diabetic retinopathy screening. The program uses algorithms to analyze retinal images and has demonstrated efficacy in improving early detection and management of diabetic retinopathy, which is crucial for reducing vision loss (162). AI models trained on retinal images can detect conditions like diabetic retinopathy, glaucoma, and AMD with high accuracy. For instance, the Singapore-based deep learning algorithm SELENA+ has been validated for its ability to detect multiple pathologies, achieving high sensitivity and specificity across several conditions (126, 163). Additionally, home-based monitoring technologies, such as smartphone apps like Alleye (164) and Home Vision Monitor (165), and portable OCT devices like Vision Home OCT (166, 167) have emerged, allowing patients with conditions like AMD to self-monitor their vision and transmit data to their healthcare providers. These innovations enhance both the quality and accessibility of eye care. Key teleophthalmology advancements are chronologically listed in Table 7.

**Table 7:** Advances in teleophthalmology.

Application	Description	Impact	Year
Remote diabetic retinopathy screening	AI-powered teleophthalmology platforms that screen for diabetic retinopathy using retinal images uploaded by patients or local clinics.	Early detection and management of diabetic retinopathy, reducing the risk of vision loss.	2018
Tele-glaucoma monitoring	Remote monitoring of intraocular pressure and optic nerve health using smart devices and AI algorithms.	Improved management of glaucoma through continuous monitoring and timely intervention.	2019
Virtual consultations	Video consultations with ophthalmologists for routine check-ups, follow-up appointments, and second opinions.	Increased access to specialist care, especially for patients in remote or underserved areas.	2020
Teleophthalmology in emergency care	Rapid remote assessment of eye injuries and acute eye conditions in emergency settings.	Quick diagnosis and treatment recommendations, reducing the need for immediate in-person visits.	2020
Home-based visual field testing	Portable devices and software allowing patients to perform visual field tests at home, with results reviewed by ophthalmologists remotely.	Convenience for patients and regular monitoring of conditions like glaucoma.	2021
Remote amblyopia treatment	A digital therapeutic platform that delivers dichoptic, eye-tracking–based amblyopia therapy via telemedicine, enabling at-home treatment with personalized video content and remote adherence monitoring.	Provides a safe and engaging alternative to traditional patching, significantly improving amblyopic eye visual acuity and stereoacuity in children while ensuring high adherence.	2021
Remote postoperative care	Monitoring and follow-up care for patients recovering from eye surgeries through telemedicine platforms.	Improved patient outcomes and convenience, reducing the need for frequent in-person visits.	2021
AI-enhanced fundus photography	Remote capture and AI analysis of fundus images to detect various retinal diseases.	Higher diagnostic accuracy and earlier detection of retinal conditions.	2022
Virtual reality training for providers	Use of virtual reality (VR) to train healthcare providers in teleophthalmology techniques and patient management.	Enhanced training outcomes, ensuring providers are well-prepared for teleophthalmology practice.	2022
Remote pediatric eye exams	Teleophthalmology tools designed specifically for pediatric patients, including interactive and child-friendly diagnostic tests conducted remotely.	Increased accessibility to eye care for children, ensuring early detection and treatment of eye conditions.	2022
Portable OCT devices	Development of portable optical coherence tomography devices that can be used in remote settings and analyzed via telemedicine platforms.	Expanded access to advanced retinal imaging for remote and underserved populations.	2022
AI-powered diagnosis for AMD	Remote evaluation and diagnosis of AMD using AI algorithms applied to retinal images.	Early detection and better monitoring of AMD progression.	2023
AI-driven predictive analytics	AI models predicting disease progression and treatment outcomes based on patient data collected remotely.	Personalized treatment plans and better disease management.	2023
Blockchain for data security	Utilizing blockchain technology to secure patient data and ensure privacy in teleophthalmology services.	Enhanced data security and patient trust in remote eye care services.	2023
Comprehensive tele-ophthalmology platforms	Integrated platforms offering a suite of diagnostic tools, patient management features, and AI-powered analysis for comprehensive eye care.	Streamlined workflows and improved patient care continuity.	2024
Smart contact lenses	Contact lenses with embedded sensors to monitor intraocular pressure and transmit data to healthcare providers remotely.	Continuous monitoring of glaucoma patients, enabling timely interventions.	2024

AMD, Age-related macular degeneration; AI, Artificial intelligence; OCT, Optical coherence tomography,

Despite these advances, teleophthalmology also brings forth multifaceted ethical, societal, and regulatory challenges that demand careful integration alongside its promising technological innovations. Ethically, ensuring robust informed consent and data security in remote environments is critical, particularly given the integration of AI algorithms that may inadvertently perpetuate biases and undermine the patient–provider relationship (168). One primary barrier is the digital divide, which can limit access to telehealth services, particularly among older adults, those with lower digital literacy, and economically disadvantaged groups (169). The COVID-19 pandemic highlighted these inequalities, as many patients struggled with the transition to remote consultations. There are also concerns regarding the depersonalization of the patient–doctor relationship, with some patients and providers reporting difficulties in building rapport through virtual platforms (170). Furthermore, technological issues, such as poor image quality due to media opacities or inadequate infrastructure, can affect the accuracy and reliability of remote diagnoses (171). Concurrently, the regulatory landscape has struggled to keep pace with these innovations, necessitating the development of updated guidelines for cross-border data transfers, licensure, cybersecurity, and reimbursement (172, 173). Addressing these interconnected issues is vital to fostering trust, ensuring equitable access, and maintaining high clinical standards, ultimately enabling teleophthalmology to fulfill its potential as an effective and ethical extension of traditional ophthalmic services. Looking to the future, several developments hold promise for overcoming these limitations. The continued refinement of AI and deep learning algorithms could automate more aspects of eye care, reducing the need for specialist input in initial screenings, which would be particularly beneficial in regions with a shortage of ophthalmologists (174). The deployment of 5G networks and advancements in data transfer technologies will also improve the speed and efficiency of teleophthalmology services, enabling real-time collaboration between healthcare providers across vast distances (175). However, the successful integration of teleophthalmology into mainstream healthcare will require robust infrastructure, significant investment in digital literacy programs, and ongoing efforts to ensure patient privacy and data security (176). In this context, virtual reality is being used to train healthcare providers in teleophthalmology techniques and patient management, enhancing training outcomes and ensuring providers are well-prepared to deliver high-quality care in a telemedicine setting (177). Meanwhile, blockchain technology is being explored for securing patient data in teleophthalmology to enhance patient trust in remote eye care services (178). Finally, economic analyses are needed to validate the cost-effectiveness of these technologies and ensure their widespread adoption (179), though initial equipment and training costs are notable barriers, but long-term savings from early detection and reduced blindness-related expenditures highlight its cost-effectiveness, particularly in remote regions (180).

## 9 Optogenetics

Optogenetics is a technique that uses light-sensitive proteins, known as opsins, to control the activity of specific cells in the retina. By introducing these opsins into retinal cells via viral vectors, researchers can make the cells responsive to light, effectively creating new photoreceptor functions in the diseased retina (181, 182). This approach is particularly promising for patients with retinitis pigmentosa, where photoreceptors are lost but inner retinal cells remain intact (Table 8) (183). Clinical trials of optogenetic therapies are currently underway, with some showing early signs of success. For instance, a Phase I/IIa trial evaluating GS030 for advanced non-syndromic retinitis pigmentosa combines gene therapy with a visual stimulation device. GS030-DP uses an AAV2.7 m8 capsid to deliver the ChrimsonR-tdTomato opsin to RGCs. ChrimsonR is notable for its red-shifted peak wavelength of 590 nm, providing enhanced safety and reduced phototoxicity compared to traditional blue light-based opsins (184, 185). The companion device, GS030-MD, emits 595 nm light pulses to activate ChrimsonR-expressing RGCs. Preliminary results suggest promising safety and partial vision recovery (186). Notably, in 2017, the FDA granted Orphan Drug Designation to GS030-MD for treating retinitis pigmentosa. In contrast, RST-001, developed by Allergan, utilizes an AAV-2 vector to deliver ChR2 to RGCs. ChR2, a Type 1 opsin, requires high-intensity blue light, which can be phototoxic (187). Despite no serious adverse events reported in a Phase I sequential dose-escalating study, the therapy’s effectiveness in improving visual function remains unproven. Additionally, it is being evaluated for dry AMD, with a Phase II study currently ongoing (182). Bionic Sight’s BS01 integrates ChronosFP gene therapy with a neuroprosthetic system. This system uses an encoder to translate visual input into brain-readable signals and a transducer to project these signals onto the retina (188). The approach aims to enhance the interaction between gene therapy and visual prosthetics, with early results indicating initial success in light and motion detection. Nanoscope Therapeutics’ vMCO-010 is an ambient light-sensitive opsin delivered via an AAV2 vector to bipolar cells. This therapy is notable for its broad activation spectrum, spanning from blue to red wavelengths, and its potential to restore vision without requiring artificial light (189, 190). Preliminary Phase I/IIa results showed significant improvements in visual acuity, and the Phase IIb trial is ongoing to further assess efficacy and safety. RST-001, GS030-MD, and vMCO-010 have all been granted Orphan Drug Designation by the FDA for the treatment of retinitis pigmentosa in 2014, 2017, and 2020, respectively (182).

**Table 8:** Clinical studies assessing effect of optogenetics in ophthalmology.

NCT ID	Phase	Condition	Intervention	Opsin	Viral vector	Company
NCT03326336	Phase I/IIa	Retinitis Pigmentosa	Drug: GS030-DP, Medical device: GS030-MD	ChrimsonR	rAAV2.7m8	GenSight Biologics
NCT02556736	Phase I/IIa	Advanced Retinitis Pigmentosa	Drug: RST-001	ChR2	rAAV2.7m8	Allergan
NCT04278131	Phase I/II	Retinitis Pigmentosa	Drug: BS01	ChronosFP	rAAV2	Bionic Sight LLC
NCT04945772	Phase IIb	Retinitis Pigmentosa	Drug: vMCO-010	MCO1	rAAV2	Nanoscope Therapeutics Inc.

Given its one-time treatment potential, preliminary data suggest potential efficacy of optogenetics, but no specific cost-effectiveness studies are available, posing a critical challenge in clinical translation of this technique (191). In the future, optimizing gene delivery methods to avoid inflammation and retinal detachment must be a key focus in optogenetics. In this context, subretinal delivery of transgenes using an AAV2 vector has proven safe and effective in treating LCA, offering a favorable biodistribution profile compared to intravitreal injections, which, while less technically challenging, may require higher viral doses and increase inflammation risks (192, 193). New delivery methods, such as suprachoroidal and sub-internal limiting membrane injections, are being investigated to potentially reduce complications and improve treatment outcomes (194).

## 10 Bionics

Bionic eyes, also known as retinal prostheses, represent an innovative approach to restoring vision in patients with severe retinal degeneration. These devices convert visual information into electrical signals that can be transmitted to the brain via surviving retinal cells or the optic nerve (195, 196). The Argus II retinal prosthesis, developed by Second Sight, was approved for use in patients with retinitis pigmentosa. The device featured a small camera mounted on glasses, which captures visual information and transmits it to an array of electrodes implanted on the retina. These electrodes stimulate the remaining retinal cells, allowing the patient to perceive light and shapes (197). However, despite its transformative potential, the Argus II was eventually discontinued. This decision was driven by several factors, including the device’s limited market size, significant financial challenges faced by Second Sight, and concerns over long-term reliability and safety. Consequently, the cessation of active technical support has raised concerns about device maintenance and the potential for adverse events in existing patients. While bionics offer limited visual resolution, they have been life-changing for some patients, enabling them to navigate their environment more independently. However, the visual experience provided by bionic eyes is still far from normal vision, and ongoing research aims to improve the resolution and functionality of these devices. For instance, the PRIMA bionic eye, developed by Pixium Vision, uses a subretinal implant with a higher density of electrodes, potentially offering improved visual resolution. Early clinical trials have shown that the PRIMA device can restore some degree of central vision in patients with geographic atrophy, a late-stage form of AMD (198). A list of bionics currently being clinically tested for various ocular conditions is provided in Table 9.

**Table 9:** Advances in bionics in ophthalmology.

Device name	Company/research consortium	Array location	Device stage	Clinical trial identifiers/status	Electrode specifications	Size	Benefits/advantages	Reported SAE/AE’s	Clinical status
NR600 System	Nano Retina, Israel (company)	Epiretinal	Clinical	NCT04295304 (recruiting)	600 3D microelectrodes (150 ± 30 μm length, 50 μm height)	Three different lengths (20–26 mm axial)	Porous, stable material; lower peak intensity; no surplus wiring; fast recovery time	Mild corneal edema, elevated IOP, intraocular lens luxation	In clinical trials in Italy, Israel, and Belgium
IMIE 256	Golden Eye Bionic, USA and IntelliMicro Medical, China (companies)	Epiretinal	Clinical	None	256 electrodes (210 μm and 160 μm diameter)	4.75 mm × 6.50 mm, 350 μm pitch	Compact size; low power consumption; biocompatible; improved manufacturability	Electrode displacement, low intraocular pressure in implanted eye	Future clinical trials planned for more patients and longer follow-up periods
POLYRETINA	Diego Ghezzi research team (École Polytechnique Fédérale de Lausanne)	Epiretinal	Pre-clinical	None	2,215 stimulating pixels (80 and 130 μm diameter)	24 mm × 24 mm × 14 mm	Foldable; reduces retinal tissue heating; large diameter photovoltaic electrodes	Low acute immune response activation	In vivo studies showed light-evoked cortical responses; tolerable after two weeks of implantation
EPI-RET3	RWTH Aachen, Germany	Epiretinal	Clinical	None	25 electrodes (100 μm diameter)	40 mm length × 3 mm width	Wireless implant; short surgical time; direct electrode to retinal stimulation	Non-progressive epiretinal gliosis; inflammatory reaction; retinal break post-implant removal; hypopyon; choroidal atrophy near tacks	Six patients were implanted for 28 days; no active clinical trials at this time
PRIMA	Pixium Vision, France (company)	Subretinal	Clinical	NCT03392324, NCT04676854 (recruiting); NCT03333954 (active, not recruiting)	378 electrodes (100 μm width)	2 × 2 mm, 30 microns thick	Minimally invasive; targeted stimulation; reduced retinal thickness	Information not available	In clinical trials for age-related macular degeneration (France and USA)
IMTC’s HARP4k Retinal prosthesis system	Iridium Medical Technology, Taiwan (company)	Subretinal	Pre-clinical	None	4,000 microelectrodes (30 μm thick)	30 mm	Wireless; high granularity; biocompatible; supports face recognition	Information not available	Designed for retinitis pigmentosa and age-related macular degeneration
Gen 2 Suprachoroidal device	Bionic Vision Australia (Research consortium)	Supra-choroidal	Clinical	NCT03406416 (completed); NCT05158049 (enrolling by invitation)	44 active electrodes (1 μm diameter)	19 × 8 mm	Decreased surgical complexity; suitable for at-home use; improvement on localization tasks	No device-related SAEs	Completed clinical trial with 4 patients with retinitis pigmentosa; suitable for long-term use in humans with retinitis pigmentosa
Phoenix-99	Bionic Vision Australia (Research consortium)	Supra-choroidal	Pre-clinical	None	98 stimulation electrodes, 1 returning electrode	18.7 × 10.8 mm, 500 μm thickness	Information not available	Corneal abrasion, ulcer, opacity; swelling; limited blinking; red eye; herniated choroid during array insertion; dislodged orbital grommet	Completed in vivo safety study
Suprachoroidal-Transretinal Stimulation (STS)	Osaka University, Japan (Research consortium)	Intrascleral	Pre-clinical	None	49 electrodes (500 μm diameter, 500 μm height)	5.8 × 5.2 × 0.5 mm	Covers large visual field; conforms to eye curvature	Moderate edema and hematomas in periorbital and head regions; conjunctival chemosis and injection observed in all cases	Completed in vivo study of wide-field dual-array STS prosthesis

Key goals for advancing bionic eyes include improving resolution, color contrast, and expanding the field of view (199, 200). Additionally, integrating visual information with the brain’s processing mechanisms presents a significant challenge, as the brain must adapt to decode the artificial signals generated by these devices. Therefore, a deeper understanding of retina-to-brain signal processing is essential for developing more effective bionic eyes (201). Despite these challenges, bionic eyes represent an exciting frontier in the quest to restore vision. As these technologies continue to evolve, they hold the potential to significantly improve the quality of life for patients with severe retinal degenerative diseases, offering new hope for those who have lost their vision.

## 11 Neuro-ophthalmology

Neuro-ophthalmology, a subspecialty that bridges neurology and ophthalmology, has seen significant advancements in recent years, particularly in developing innovative treatments for optic nerve and visual pathway disorders. These innovations aim to restore vision by bypassing damaged ocular structures and directly stimulating the visual pathways (Table 10) (202). One of the most promising developments is the Intracortical Visual Prosthesis (ICVP), which aims to restore vision by interfacing directly with the visual cortex. This technology involves implanting intracortical electrode arrays that stimulate the visual cortex in response to external visual inputs (203). Early experimental studies have focused on the safety and biocompatibility of these implants, with some showing potential for restoring basic visual functions such as light perception and shape recognition in patients with severe visual impairments (204, 205). Another innovative project in neuro-ophthalmology is Cortivis, which seeks to develop a cortical visual prosthesis similar to the ICVP. Cortivis involves implanting electrodes in the visual cortex to bypass damaged retinal or optic nerve pathways (206). It utilizes the Utah Electrode Array, which consists of 100 electrodes with lengths ranging from 1.0 to 1.5 mm (207). Currently in the preclinical stage, Cortivis plans to initiate human trials soon. If successful, it could provide a new treatment option for patients with conditions like retinitis pigmentosa or optic nerve atrophy, where traditional treatments are ineffective (208). The Orion project, developed by Second Sight, is one of the most advanced cortical visual prostheses. Orion bypasses the eye and optic nerve entirely, directly stimulating the visual cortex to restore some degree of vision in blind individuals (209, 210). Clinical trials have demonstrated that Orion can provide basic visual cues, such as the perception of light and simple shapes, in patients who have lost their vision due to retinal or optic nerve damage (211). In a study involving 5 blind and 15 sighted patients, researchers tested Orion electrodes implanted in the visual cortex to stimulate vision. They compared two fixation methods: unimanual fixation (using one finger) and bimanual fixation (overlaying one finger on the other). The study found that bimanual fixation combined with relative mapping provided the most accurate estimates of phosphene organization (212). These promising results have led to FDA-approved clinical trials in the United States, marking a significant milestone in the development of cortical visual prostheses (211). The Artificial Vision by Direct Optic Nerve Electrode (AV-DONE) system is another notable development. It involves an electrode device implanted into the optic disc. During trials, patients underwent electrical stimulation sessions 9 and 23 months after implantation. Over 50% of the tests successfully produced phosphene perceptions, with thresholds identified by the stimulation current. Ophthalmologic exams conducted before implantation and every 6 months during a 25-month follow-up revealed no severe complications. The device was found to reduce surgical time, minimize damage to optic nerve fibers, and allow for more electrodes to be fixed compared to earlier devices, indicating its safety and potential benefits for future patients (213).

**Table 10:** Neuro-ophthalmology advances in ophthalmology.

Device name	Company/research consortium	Array location	Device stage	Clinical trial identifiers/status	Electrode specifications	Size	Benefits/advantages	Reported SAE/AE’s	Clinical status
ORION	Second Sight Medical Products, USA (company)	Occipital lobe	Clinical	NCT03344848 (active, not recruiting)	60 electrodes	Information not available	Treats a wider variety of diseases; neural placement; ability to navigate environment	Seizure, bilateral hand twitch, headache, visual aura, visual phenomenon	Ongoing clinical trials evaluating the safety of the device and surgery
CORTIVIS	Biomedical Technologies, Spain (company)	Occipital lobe	Clinical	NCT02983370 (recruiting)	100 electrodes (1.0–1.5 mm length)	4 mm × 4 mm base	Information not available	Information not available	In clinical trial for severe visual impairment with bilateral visual loss
Intracortical Visual Prosthesis (ICVP)	Illinois Institute of Technology, USA	Visual cortex	Clinical	NCT04634383 (active, recruiting)	16 electrodes per module	2 mm × 2 mm	Wireless; camera images communicated directly to brain	Information not available	In clinical trials to test safety and feasibility of eliciting visual percepts in response to electrical stimulation in persons with blindness
Artificial Vision by Direct Optic Nerve Electrode (AV-DONE)	NIDEK CO, Japan (company)	Optic nerve	Pre-clinical	None	7 stimulation electrodes (50 μm diameter)	Rod: 100 μm diameter; cylindrical board: 2.0 mm diameter	Easy access to optic nerve; stimulates wide visual field; elicits small to large phosphenes	Information not available	Clinical study completed for 1 patient with retinitis pigmentosa

Despite these advancements, several challenges remain. The complexity of visual pathways and the brain’s processing of visual information pose significant hurdles to developing effective cortical prostheses (196, 214). The Genneris project addresses this by integrating brain-computer interface (BCI) technology with personalized visual prosthetics (215). The Genneris system includes a headband with a camera, a visual processing device with software, a wireless transmitter, and brain tiles measuring 9 mm × 9 mm. The camera captures video, which is processed to extract essential information, and this data is wirelessly transmitted to the implanted brain tiles. The tiles generate electrical pulses that stimulate the brain through a microelectrode array, creating visual patterns with up to 473 light dots (phosphenes). This system provides crucial information about environments and the presence of people and objects. It can transmit data to up to 10 implants, each stimulating 43 areas of the visual cortex (215, 216). By tailoring visual prosthetics to individual needs, Genneris aims to enhance the efficacy of neuroprosthetic devices. While still in development, Genneris has the potential to improve artificial vision quality in patients with severe visual impairments (215). Overall, as these neuro-ophthalmology-based technologies advance, they hold the potential to transform the treatment of optic nerve and visual pathway disorders, offering new hope to patients with previously untreatable conditions.

## 12 Conclusion

The landscape of ophthalmology is undergoing rapid transformation, driven by cutting-edge technological advancements. Gene and stem cell therapies are leading this revolution, with gene therapy correcting genetic defects and stem cell therapy offering potential for retinal regeneration. These approaches have already demonstrated significant promise, particularly for inherited retinal diseases such as retinitis pigmentosa and LCA. Therapeutic advancements, including anti-VEGF therapies, continue to improve the management of conditions like AMD and DME, with newer treatments providing longer-lasting effects and reducing the treatment burden. Nanotechnology is playing a crucial role in enhancing drug delivery systems, ensuring more targeted and effective treatments for ocular diseases. Advanced imaging techniques, supported by AI, are enhancing early diagnosis, disease monitoring, and personalized treatment planning. AI’s integration into teleophthalmology is expanding access to care, particularly in remote and/or underserved regions, offering new opportunities for timely and efficient eye care. Emerging technologies such as optogenetics and bionic eyes hold exciting potential for restoring vision in patients with severe retinal degeneration, while innovations in neuro-ophthalmology, such as cortical visual prostheses, could restore vision in patients with optic nerve damage. However, significant challenges remain, including issues related to cost, safety, and long-term efficacy. As these technologies mature, their potential to address previously untreatable conditions grows, offering new hope to millions affected by visual impairment. Continued research, collaboration, and refinement of these therapies will be essential in realizing the full potential of these breakthroughs, ultimately bringing us closer to a future where vision loss is no longer inevitable.

## Glossary

3D: Three-dimensional
AAVs: Adeno-associated viruses
ABMSCs: Autologous bone marrow mesenchymal stem cells
ADMSCs: Adipose-derived MSCs
AI: Artificial intelligence
AMD: Age-related macular degeneration
AO: Adaptive optics
AOSLO: AO with scanning laser ophthalmoscopy
AUC: Areas under the curve
AV-DONE: Artificial Vision by Direct Optic Nerve Electrode
BCI: Brain-computer interface
cGVHD: Chronic graft-versus-host disease
CLET: Cultivated limbal epithelium transplantation
CNGA3: Cyclic nucleotide-gated cation channel alpha-3
CNGB3: Cyclic nucleotide-gated cation channel beta-3
CNNs: Convolutional neural networks
cSLO: Confocal scanning laser ophthalmoscopy
DED: Dry eye disease
DME: Diabetic macular edema
FA: Fluorescein angiography
FDA: Food and Drug Administration
hESC: Human embryonic stem cell
ICG: Indocyanine green angiography
ICVP: Intracortical Visual Prosthesis
IOP: Intraocular pressure
LCA: Leber congenital amaurosis
LSCD: Limbal stem cell deficiency
LSCs: Limbal stem cells
mfERG: Multifocal electroretinography
MSCs: Mesenchymal stem cells
OCT: Optical coherence tomography
OCTA: Optical coherence tomography angiography
RGCs: Retinal ganglion cells
RPCs: Retinal progenitor cells
RPE: Retinal pigment epithelium
RPGR: Retinitis pigmentosa GTPase regulator
SLET: Simple limbal epithelial transplantation
SS-OCT: Swept-source optical coherence tomography
STGD1: Stargardt disease type 1
TGF-β: Transforming growth factor-beta
TMSCs: Trabecular meshwork stem cells
UBM: Ultrasound biomicroscopy
VEGF: Vascular endothelial growth factor
